# Post-mortem investigation of deaths due to pneumonia in children aged 1–59 months in sub-Saharan Africa and South Asia from 2016 to 2022: an observational study

**DOI:** 10.1016/S2352-4642(23)00328-0

**Published:** 2024-03

**Authors:** Sana Mahtab, Dianna M Blau, Zachary J Madewell, Ikechukwu Ogbuanu, Julius Ojulong, Sandra Lako, Hailemariam Legesse, Joseph S Bangura, Quique Bassat, Inacio Mandomando, Elisio Xerinda, Fabiola Fernandes, Rosauro Varo, Samba O Sow, Karen L Kotloff, Milagritos D Tapia, Adama Mamby Keita, Diakaridia Sidibe, Dickens Onyango, Victor Akelo, Dickson Gethi, Jennifer R Verani, Gunturu Revathi, J Anthony G Scott, Nega Assefa, Lola Madrid, Hiwot Bizuayehu, Tseyon Tesfaye Tirfe, Shams El Arifeen, Emily S Gurley, Kazi Munisul Islam, Muntasir Alam, Mohammad Zahid Hossain, Ziyaad Dangor, Vicky L Baillie, Martin Hale, Portia Mutevedzi, Robert F Breiman, Cynthia G Whitney, Shabir A Madhi, CHAMPS Consortium, Yasmin Adam, Janet Agaya, A.S.M. Nawshad Uddin Ahmed, Dilruba Ahmed, Addisu Alemu, Solomon Ali, Soter Ameh, George Aol, Solveig Argeseanu, Farida Ariuman, Oluseyi Balogun, Sanwarul Bari, Margaret Basket, Ferdousi Begum, Manu Bhandari, John Blevins, James Bunn, Courtney Bursuc, Carrie Jo Cain, Richard Chawana, Kiranpreet Chawla, Cornell Chukwuegbo, Kounandji Diarra, Tiéman Diarra, Maureen Diaz, Babatunde Duduyemi, Karen D. Fairchild, Meerjady Sabrina Flora, Ashleigh Fritz, Mischka Garel, Brigitte Gaume, Mahlet Abayneh Gizaw, Nelesh P. Govender, Carol L. Greene, Tadesse Gure, Binyam Halu, Mahbubul Hoque, Cleopas Hwinya, Alexander M. Ibrahim, Kitiezo Aggrey Igunza, Ferdousi Islam, Okokon Ita, Amara Jambai, J. Kristie Johnson, Jane Juma, Erick Kaluma, Mohammed Kamal, Osman Kaykay, Sartie Kenneh, Sammy Khagayi, Rima Koka, Diakaridia Kone, Jeffrey P. Koplan, Nana Kourouma, Dickens Kowuor, Kristin LaHatte, Sanjay G. Lala, Kyu Han Lee, Lucy Liu, Hennie Lombaard, Maria Maixenchs, Zara Manhique, Margaret Mannah, Roosecelis Martines, Ronald Mash, Ashka Mehta, Clara Menéndez, Thomas Misore, Sibone Mocumbi, Andrew Moseray, Francis Moses, Christopher Muga, Khátia Munguambe, Nellie Myburgh, Shailesh Nair, Pedzisai Ndagurwa, Ariel Nhacolo, Tacilta Nhampossa, Princewill Nwajiobi, Christine Ochola, Richard Oliech, Bernard Oluoch, Uma U. Onwuchekwa, Peter Nyamthimba Onyango, Stian MS Orlien, Peter Otieno, Joseph Oundo, Harun Owuor, Shahana Parveen, Karen Petersen, Samuel Pratt, Mahbubur Rahman, Mohammad Mosiur Rahman, Mustafizur Rahman, Sarah Raymer, Jana Ritter, Navit T. Salzberg, Solomon Samura, Sulaiman Sannoh, Doh Sanogo, Martin Seppeh, Tom Sesay, Joseph Kamanda Sesay, Tahmina Shirin, Seydou Sissoko, Francis Smart, Gillian Sorour, James Squire, Alim Swaray-Deen, Peter J. Swart, Fatmata Bintu Tarawally, Saria Tasnim, Fikremelekot Temesgen, Sharon M. Tennant, Cheick Bougadari Traore, Awa Traore, Sithembiso Velaphi, Kurt Vyas, Ashutosh Wadhwa, Jeannette Wadula, Jessica Waller, Valentine Wanga, Shamta Warang, Joyce Akinyi Were, Tais Wilson, Jonas Winchell, Amy Wise, Jakob Witherbee, Melisachew Mulatu Yeshi, K. Zaman

**Affiliations:** a South African Medical Research Council Vaccines and Infectious Diseases Analytics Research Unit, Faculty of Health Sciences, University of the Witwatersrand, Johannesburg, South Africa; b Global Health Center, Centers for Disease Control and Prevention, Atlanta, GA, USA; c National Center for Immunization and Respiratory Disease, Centers for Disease Control and Prevention, Atlanta, GA, USA; d Crown Agents, Freetown, Sierra Leone; e ICAP – Columbia University, Makeni, Sierra Leone; f Aberdeen Women's Centre, Freetown, Sierra Leone; g UNICEF Sierra Leone, Freetown, Sierra Leone; h Caritas International-Sierra Leone, Freetown, Sierra Leone; i Centro de Investigação em Saúde de Manhiça, Maputo, Mozambique; j ISGlobal - Hospital Clínic, Unversitat de Barcelona, Barcelona, Spain; k Institutó Catalana de Recerca I Estudis Avançats, Barcelona, Spain; l Pediatrics Department, Hospital Sant Joan de Déu, Universitat de Barcelona, Esplugues, Barcelona, Spain; m Consorcio de Investigación Biomédica en Red de Epidemiología y Salud Pública, Madrid, Spain; n Instituto Nacional de Saúde, Maputo, Mozambique; o Department of Pathology, Maputo Central Hospital, Maputo, Mozambique; p Centre pour le Développement des Vaccins, Ministère de la Santé, Bamako, Mali; q Department of Pediatrics and Department of Medicine, Center for Vaccine Development and Global Health, University of Maryland School of Medicine, Baltimore, MD, USA; r Kisumu County Department of Health, Kisumu, Kenya; s Centers for Disease Control and Prevention—Kenya, Kisumu, Kenya; t Kenya Medical Research Institute-Center for Global Health Research (KEMRI-CGHR), Kisumu, Kenya; u Department of Pathology, Aga Khan University, Nairobi, Kenya; v Department of Infectious Disease Epidemiology, London School of Hygiene & Tropical Medicine, London, UK; w College of Health and Medical Sciences, Haramaya University, Harar, Ethiopia; x Department of Microbiology, Addis Ababa Burn, Emergency and Trauma Hospital, Addis Ababa, Ethiopia; y International Center for Diarrhoeal Diseases Research, Dhaka, Bangladesh; z Department of Epidemiology, Johns Hopkins Bloomberg School of Public Health, Baltimore, MD, USA; aa National Health Laboratory Service, Department of Anatomical Pathology, School of Pathology, Faculty of Health Sciences, University of the Witwatersrand, Johannesburg, South Africa; ab Emory Global Health Institute, Emory University, Atlanta, Georgia, USA; ac Wits Infectious Diseases and Oncology Research Institute, University of the Witwatersrand, Faculty of Health Sciences, Johannesburg, South Africa

## Abstract

**Background:**

The Child Health and Mortality Prevention Surveillance (CHAMPS) Network programme undertakes post-mortem minimally invasive tissue sampling (MITS), together with collection of ante-mortem clinical information, to investigate causes of childhood deaths across multiple countries. We aimed to evaluate the overall contribution of pneumonia in the causal pathway to death and the causative pathogens of fatal pneumonia in children aged 1–59 months enrolled in the CHAMPS Network.

**Methods:**

In this observational study we analysed deaths occurring between Dec 16, 2016, and Dec 31, 2022, in the CHAMPS Network across six countries in sub-Saharan Africa (Ethiopia, Kenya, Mali, Mozambique, Sierra Leone, and South Africa) and one in South Asia (Bangladesh). A standardised approach of MITS was undertaken on decedents within 24–72 h of death. Diagnostic tests included blood culture, multi-organism targeted nucleic acid amplifications tests (NAATs) of blood and lung tissue, and histopathology examination of various organ tissue samples. An interdisciplinary expert panel at each site reviewed case data to attribute the cause of death and pathogenesis thereof on the basis of WHO-recommended reporting standards.

**Findings:**

Pneumonia was attributed in the causal pathway of death in 455 (40·6%) of 1120 decedents, with a median age at death of 9 (IQR 4–19) months. Causative pathogens were identified in 377 (82·9%) of 455 pneumonia deaths, and multiple pathogens were implicated in 218 (57·8%) of 377 deaths. 306 (67·3%) of 455 deaths occurred in the community or within 72 h of hospital admission (presumed to be community-acquired pneumonia), with the leading bacterial pathogens being *Streptococcus pneumoniae* (108 [35·3%]), *Klebsiella pneumoniae* (78 [25·5%]), and non-typeable *Haemophilus influenzae* (37 [12·1%]). 149 (32·7%) deaths occurred 72 h or more after hospital admission (presumed to be hospital-acquired pneumonia), with the most common pathogens being *K pneumoniae* (64 [43·0%]), *Acinetobacter baumannii* (19 [12·8%]), *S pneumoniae* (15 [10·1%]), and *Pseudomonas aeruginosa* (15 [10·1%]). Overall, viruses were implicated in 145 (31·9%) of 455 pneumonia-related deaths, including 54 (11·9%) of 455 attributed to cytomegalovirus and 29 (6·4%) of 455 attributed to respiratory syncytial virus.

**Interpretation:**

Pneumonia contributed to 40·6% of all childhood deaths in this analysis. The use of post-mortem MITS enabled biological ascertainment of the cause of death in the majority (82·9%) of childhood deaths attributed to pneumonia, with more than one pathogen being commonly implicated in the same case. The prominent role of *K pneumoniae*, non-typable *H influenzae*, and *S pneumoniae* highlight the need to review empirical management guidelines for management of very severe pneumonia in low-income and middle-income settings, and the need for research into new or improved vaccines against these pathogens.

**Funding:**

Bill & Melinda Gates Foundation.

## Introduction

Globally, pneumonia is the leading infectious cause of death in children younger than 5 years.^1^ In 2019, pneumonia was estimated to have caused 740 180 deaths in this age group. Approximately 80% of childhood deaths due to pneumonia occur in South Asia and sub-Saharan Africa.^1^ Current estimates of the causes of childhood deaths are primarily based only on the condition considered to have underpinned the pathway to death (ie, the underlying cause), to ensure that the cumulative cause-specific number of deaths aligns with overall age-specific number of decedents.^2^ Consequently, there could be inadvertent underestimation of the role of pneumonia in childhood deaths, should pneumonia be implicated in the casual pathway but not attributed as the underlying cause. Furthermore, current modelling of attributable causes of childhood deaths is based on vital registration and verbal autopsy records,^3^ which are generally unable to make pathogen-specific diagnoses for infectious causes.^4^ More granular information on pathogen-specific causes of death in children could inform strategies to reduce under-5 mortality rates, which were estimated at 38 deaths per 1000 livebirths in 2021, to reach the UN Sustainable Development Goal 3.2 target of 25 deaths per 1000 livebirths by 2030.^5^

**Research in context**

**Evidence before this study**

Pneumonia is the leading cause of death due to infectious diseases in children younger than 5 years. Determining the cause of pneumonia is challenging, as direct lung tissue sampling is uncommon ante-mortem and post-mortem in low-income and middle-income settings. We did a systematic literature search of PubMed for papers published in English from database inception up to Dec 31, 2022, that evaluated the causes of fatal pneumonia in children younger than 5 years using the following search terms and research strategy: “pneumonia” OR “lower respiratory tract infection” AND “cause of death” AND “childhood” AND “etiology” OR “pathogen” AND “diagnostic autopsy”. The initial search yielded 37 results. Through a review of the title and abstract, excluding case studies on children with cancer, congenital anomalies, and other non-pneumonia deaths, we identified 12 studies that reported on the causes of pneumonia in children. Only nine of these 12 studies reported on specific pathogenic organisms, all of which were conducted at a single site or one country. Enhancing post-mortem examinations in low-resource settings, particularly sub-Saharan Africa with its substantial respiratory disease burden, is crucial, but hindered by resource limitations, technical capacity, misinformation, cultural considerations, and stigma around conventional autopsies.

**Added value of this study**

Our study provides in-depth information about the overall contribution of pneumonia and the cause thereof in decedents aged 1–59 months across six countries in sub-Saharan Africa and one in South Asia. We used post-mortem minimally invasive tissue sampling (MITS) together with ante-mortem clinical data to quantify the overall role of pneumonia in the causal pathway to childhood death and ascertain pathogen-specific causes of pneumonia. Pneumonia was implicated in the causal pathway of death in 40·6% of decedents. Of all fatal pneumonia cases, 67·3% occurred in the community or within 72 h of hospital admission (presumed to be community-acquired) and 32·7% occurred at least 72 h after admission to hospital (presumed to be hospital-acquired). More than one pathogen was implicated in the pathogenesis of 57·8% of fatal pneumonia cases. Overall, co-infections included bacterial-bacterial (70·6%), bacterial-viral (37·2%), bacterial-fungal (11·9%), fungal-viral (7·8%), and viral-viral (7·3%) combinations. Overall, *Klebsiella pneumoniae, Streptococcus pneumoniae*, and non-typable *Haemophilus influenzae* were the most common bacterial pathogens; cytomegalovirus, respiratory syncytial virus, and *Pneumocystis jirovecii* were the most common non-bacterial pathogens attributed to pneumonia-associated deaths. In presumed community-acquired pneumonia deaths, the most commonly implicated bacterial pathogens were *S pneumoniae, K pneumoniae*, non-typable *H influenzae*, and *Staphylococcus aureus*, while the most common viruses were cytomegalovirus and respiratory syncytial virus. Notably, there were no pneumonia-associated deaths attributed to *Mycobacterium tuberculosis.*

**Implications of all the available evidence**

The high prevalence of co-infections among pneumonia-related deaths involving both bacterial and viral pathogens underscores the complexity of the causes of severe pneumonia, emphasising the need to consider multiple causative agents in diagnostic and treatment strategies for severe pneumonia. The biological confirmation of the dominance of *K pneumoniae, S pneumoniae*, and non-typable *H influenzae* highlights which organisms should be targeted for future interventions, including improvement of current or development of new vaccines. Similarly, the attribution of childhood pneumonia deaths to viruses and fungi indicates the need to review empirical management strategies for severe pneumonia. By incorporating our study findings into decision making for global health agendas, policy makers and stakeholders can design evidence-based interventions, prioritise resource allocation, and advance research efforts to reduce childhood mortality in high-burden regions.

Post-mortem biological characterisation for specific causes of childhood deaths, including pneumonia, is inadequate, particularly in low-income and middle-income settings. Furthermore, in the absence of direct ante-mortem sampling of lung tissue, current diagnostic tools do not have sufficient sensitivity and specificity for diagnosing the cause of pneumonia, especially bacterial pathogens. Consequently, estimates of pathogen-specific causes of deaths due to pneumonia rely on imputation from ante-mortem epidemiological and vaccine-probe studies, primarily focusing on vaccine-preventable pathogens.^6^

Complete diagnostic autopsy, which is considered the gold standard for determining the cause of death, is rarely performed in many settings due to cultural, economic, and logistical factors. The Child Health and Mortality Prevention Surveillance (CHAMPS) Network uses post-mortem minimally invasive tissue sampling (MITS), which provides comparable findings to complete diagnostic autopsy in attributing the cause of death due to infectious causes.^6^ Post-mortem MITS enables comprehensive examination of microbiology and pathology and has been used to investigate causes of childhood deaths, including characterisation of pathogen-specific causes of infection-related cases.^7^, ^8^, ^9^

In this observational study, we aimed to analyse the overall contribution of pneumonia in the causal pathway to death, and pathogen-specific causes of pneumonia, in decedents aged 1–59 months in the multi-country CHAMPS Network programme. We included all enrolled cases in CHAMPS on which the Determination of Cause of Death (DeCoDe) process had been completed from Dec 16, 2016, to Dec 31, 2022. This analysis complements an earlier report on the causes of childhood deaths in CHAMPS, which analysed data from Dec 16, 2016, to Dec 31, 2020, in which pathogen-specific causes of pneumonia-associated deaths were not reported.^10^

## Methods

### Study design and participants

Details of the CHAMPS methodology have been previously published.^7^, ^11^, ^12^ The surveillance is conducted in defined catchment areas across six countries in sub-Saharan Africa (Ethiopia, Kenya, Mali, Mozambique, Sierra Leone, and South Africa) and one country in South Asia (Bangladesh). The site characteristics and inclusion criteria for MITS have been previously reported.^11^ Briefly, CHAMPS surveillance aims to identify stillbirths and deaths in children younger than 5 years residing in the catchment areas and aims to include deaths occurring in the community or at health-care facilities (appendix p 26).

The ethics committees at each site and at Emory University (Atlanta, GA, USA) approved overall and site-specific protocols (Emory Institutional Review Board: 00091706). CHAMPS protocols are available online. Parents or legal guardians provided written informed consent for the decedents' inclusion before any MITS procedure was undertaken.

### Procedures

The MITS is done within 24 h of death, with a possible extension to 72 h if refrigeration of the decedent was done shortly after death. Standardised procedures are followed for collecting tissue specimens from multiple sites in the lung, liver, and brain, blood and cerebrospinal fluid (CSF), and nasopharyngeal and rectal swabs from all decedents (appendix p 26).^13^ Testing of samples includes cultures of blood and CSF for bacteria, nucleic acid amplification tests (NAATs) on blood samples for HIV-1, testing for *Mycobacterium tuberculosis* with Cepheid GeneXpert (Sunnyvale, CA, USA), and malaria smear and rapid antigen tests on blood samples. Organ tissue specimens undergo routine histopathology, special stains, and immunohistochemistry if indicated.^12^ Multiplexed TaqMan Array Cards, which target 126 organisms, are used for real-time PCR testing. There are 47 target organisms, including SARS-CoV-2, on the TaqMan Array Cards used for testing of lung tissue samples (appendix pp 2–3). Ante-mortem clinical information is abstracted from medical records, and standardised verbal autopsies are conducted by trained medical staff with the consent of the parent or guardian.^7^

A site-specific DeCoDe panel, comprising specialists in paediatrics, histopathology, microbiology, obstetrics, infectious diseases, and epidemiology, reviewed all information for each decedent.^7^ The DeCoDe panel followed WHO International Classification of Diseases, 10th Revision (ICD-10) recommendation on reporting of causes of death. The cause of death reporting includes ascertaining the condition considered to have precipitated the chain of events leading to the death (ie, the underlying cause). Furthermore, the condition considered to have finally caused the death (ie, the immediate cause), as well as any additional clinically significant condition occurring between the onset of the underlying disease and before the immediate cause of death (ie, the antecedent condition), were ascertained by the DeCoDe panel.^14^ The DeCoDe panels attributed deaths to pneumonia and specific pathogenic causes of pneumonia and other conditions in the causal pathway according to diagnosis standards developed by CHAMPS (appendix pp 4–10). The strength of evidence was graded in three levels; for pneumonia, level 1 (the highest level of certainty) required lung tissue histopathology evidence of pyogenic pneumonia or pre-mortem infiltrate or pleural effusion accompanied by documented pneumonia symptoms. Deaths presumed to be caused by pneumonia but with less specific evidence were assigned level 2 (moderate evidence) or level 3 (weakest evidence), as detailed in the appendix (pp 4–10) and the abovementioned diagnosis standards developed by CHAMPS.

### Statistical analysis

For this report, we analysed all cases where pneumonia was attributed by the DeCoDe panel to be in the causal pathway of death, irrespective of whether it was an underlying, immediate, or antecedent condition. We excluded cases of pneumonitis attributed to aspiration of food or vomitus. The analyses were also stratified by age groups of 1 months to younger than 6 months (early infancy), 6 months to younger than 12 months (late infancy), and 12–59 months (childhood). We also did site-specific analyses. All pathogens adjudicated by the DeCoDe panel as contributing to pneumonia were included for an individual case. Consequently, the number of pathogens reported could exceed the total number of decedents. Additionally, the diagnosis of pneumonia could feature more than once in the causal pathway of death, if separate episodes were attributed by the DeCoDe panel. As an example, a child admitted with pneumonia due to respiratory syncytial virus could have subsequently died from a hospital-acquired infection resulting in pneumonia.

Descriptive statistics were calculated, providing medians with IQRs for continuous variables and proportions for categorical variables. Categorical variables were assessed with Pearson's χ^2^ test. Continuous variables were compared with the Mann-Whitney *U* test for two-group comparisons and the Kruskal-Wallis rank sum test for comparisons involving more than two groups. Malnutrition at the time of death was characterised with WHO Child Growth Standards to calculate Z scores for bodyweight-for-age as a measure of undernutrition, length-for-age as a measure of stunting, and bodyweight-for-length as a measure of wasting. Z scores were categorised as normal (≥–2 SD), moderate (<–2 SD to –3 SD), and severe (<–3 SD). We stratified pneumonia-related deaths on the basis of whether they occurred in the community or within 72 h of hospital admission (presumed community-acquired pneumonia) or whether they occurred at least 72 h after hospital admission (presumed hospital-acquired pneumonia). We also conducted a sensitivity analysis defining deaths from presumed hospital-acquired pneumonia as deaths that occurred at least 120 h after hospital admission to examine whether the definition affects our findings. Statistical analyses were conducted with R software (version 4.2.3).

### Role of the funding source

The funder participated in discussions on study design and data collection. The funder did not participate in the conduct or management of the study; analysis or interpretation of data; preparation, review, or approval of the manuscript; or the decision to submit the manuscript for publication.

## Results

We analysed data from 1120 decedents enrolled during the study period (appendix pp 11, 27). Pneumonia was in the causal pathway of death for 455 (40·6%) of 1120 decedents; it was the underlying cause in 105 (9·4%) of 1120, the antecedent cause in 201 (17·9%) of 1120, and the immediate cause in 164 (14·6%) of 1120. In 15 decedents, more than one pneumonia episode was implicated in the causal pathway to death. The median age of children who died from pneumonia was 9 months (IQR 4–19) and 265 (58·2%) were male (table 1). The ethnicity profile of decedents is reported in the appendix (p 12). Pneumonia was present in the causal pathway of death for 170 (47·4%) of 359 deaths in the young infancy group, 94 (41·2%) of 228 deaths in the late infancy group, and 191 (35·8%) of 533 deaths in the childhood age group. The site-specific age group distribution of decedents with and without pneumonia in the causal pathway is reported in the appendix (p 28).

**Table 1:** Characteristics of decedents aged 1–59 months stratified by whether pneumonia was or was not attributed in the causal pathway to death in the CHAMPS Network, from Dec 16, 2016, to Dec 31, 2022

		**Overall (N=1120)**	Deaths from causes other than pneumonia (N=665)*	**Deaths attributed to pneumonia (N=455)**†	**p value**
Age at death					0·003
	Early infants (1 month to <6 months)	359 (32·1%)	189 (28·4%)	170 (37·4%)	..
	Late infants (≥6 months to <12 months)	228 (20·4%)	134 (20·2%)	94 (20·7%)	..
	Children (≥12 months to <60 months)	533 (47·6%)	342 (51·4%)	191 (42·0%)	..
Median age (IQR), months		11 (4–23)	12 (5–26)	9 (4–19)	<0·0001
Sex‡					0·081
	Female	504 (45·0%)	314 (47·2%)	190 (41·8%)	..
	Male	616 (55·0%)	351 (52·8%)	265 (58·2%)	..
HIV status					<0·0001
	HIV-positive	118 (10·5%)	47 (7·1%)	71 (15·6%)	..
	HIV-negative infant exposed to HIV	144 (12·9%)	84 (12·6%)	60 (13·2%)	..
	HIV-negative with unknown HIV exposure§	858 (76·6%)	534 (80·3%)	324 (71·2%)	..
Bodyweight for height Z score					0·006
	Normal bodyweight for height (≥–2 SD)	484 (43·2%)	309 (46·5%)	175 (38·5%)	..
	Moderate wasting (<–2 SD to −3 SD)	155 (13·8%)	99 (14·9%)	56 (12·3%)	..
	Severe wasting (<–3 SD)	407 (36·3%)	218 (32·8%)	189 (41·5%)	..
	Not recorded	74 (6·6%)	39 (5·9%)	35 (7·7%)	..
Bodyweight for age Z score					0·0004
	Normal bodyweight for age (≥–2SD)	456 (40·7%)	302 (45·4%)	154 (33·8%)	..
	Moderate underweight (<–2 SD to −3 SD)	171 (15·3%)	103 (15·5%)	68 (14·9%)	..
	Severe underweight (<–3 SD)	476 (42·5%)	250 (37·6%)	226 (49·7%)	..
	Not recorded	17 (1·5%)	10 (1·5%)	7 (1·5%)	..
Height for age Z score					0·005
	Normal height for age (≥–2SD)	659 (58·8%)	414 (62·3%)	245 (53·8%)	..
	Moderate stunting (<–2 SD to −3 SD)	160 (14·3%)	98 (14·7%)	62 (13·6%)	..
	Severe stunting (<– 3 SD)	296 (26·4%)	151 (22·7%)	145 (31·9%)	..
	Not recorded	5 (0·4%)	2 (0·3%)	3 (0·7%)	..
Median (IQR) time between death and MITS collection, h		14 (7–21)	14 (7–21)	14 (7–22)	0·50
Location of death					0·015
	Community	288 (25·7%)	189 (28·4%)	99 (21·8%)	..
	Health facility	832 (74·3%)	476 (71·6%)	356 (78·2%)	..
For health facility deaths¶					
	Median number of days between admission and death	1 (0–6)	1 (0–5)	2 (1–8)	<0·0001
Sites					<0·0001
	Bangladesh	6 (0·5%)	4 (0·6%)	2 (0·4%)	..
	Ethiopia	26 (2·3%)	6 (0·9%)	20 (4·4%)	..
	Kenya	270 (24·1%)	192 (28·9%)	78 (17·1%)	..
	Mali	60 (5·4%)	30 (4·5%)	30 (6·6%)	..
	Mozambique	191 (17·1%)	101 (15·2%)	90 (19·8%)	..
	Sierra Leone	283 (25·3%)	199 (29·9%)	84 (18·5%)	..
	South Africa	284 (25·4%)	133 (20·0%)	151 (33·2%)	..

Data are n (%) or median (IQR). CHAMPS=Child Health and Mortality Prevention Surveillance. MITS=minimally invasive tissue sampling.

* Denominator was n=453 for median days between hospital admission and death.

† Denominator was n=332 for median days between hospital admission and death.

‡ Sex was determined by parent report or medical records.

§ Data were missing on confirmed lack of exposure to HIV, but these children all tested HIV negative therefore we reported these deaths as HIV negative.

¶ The number of deaths that occurred in health facilities (n=832) was used as the denominator to calculate the median number of days between admission and death.

Among deaths that occurred in the hospital, the median time from admission to death was 2 days (IQR 1–8). 306 (67·3%) of 455 deaths attributed to pneumonia were classified as presumed community-acquired pneumonia and 149 (32·7%) of 455 were classified as presumed hospital-acquired pneumonia. The MITS was performed a median of 14 h (IQR 7–21) after death. 406 (89·2%) of 455 pneumonia diagnoses were graded as level 1 certainty, 38 (8·4%) of 455 were graded as level 2 certainty, and eight (1·8%) of 455 were graded as level 3 certainty, while there was no grading for three cases. Of the 455 decedents with pneumonia in the causal pathway, 151 (33·2%) were from South Africa, 90 (19·8%) were from Mozambique, 84 (18·5%) were from Sierra Leone, 78 (17·1%) were from Kenya, 30 (6·6%) were from Mali, 20 (4·4%) were from Ethiopia, and two (0·4%) from Bangladesh. The overall proportion of all deaths attributed to pneumonia was highest in Ethiopia (20 [76·9%] of 26) and South Africa (151 [53·2%] of 284), and lowest in Kenya (78 [28·9%] of 270). Pneumonia-attributed deaths occurring outside of health facilities were most common in Kenya (36 [46·2%] of 78) and Ethiopia (ten [50·0%] of 20) and least common in Sierra Leone (six [7·1%] of 84; appendix p 13).

**Figure 1:**
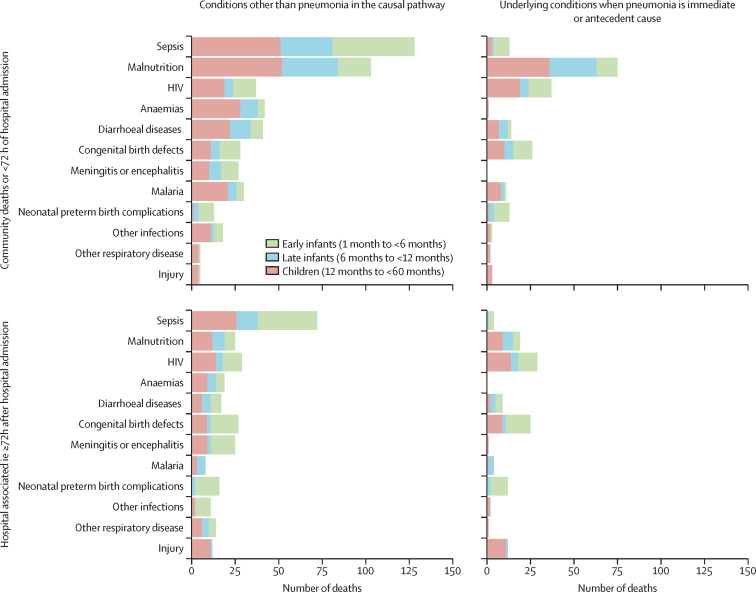
Common conditions other than pneumonia that were in the causal pathway, based on CHAMPS Network data for Dec 16, 2016, to Dec 31, 2022 Most common conditions other than pneumonia that were in the causal pathway leading to death for pneumonia, by age group and by whether the death from pneumonia occurred in the community or in less than 72 h of hospital admission or whether it occurred at least 72 h after hospital admission. The left panels include other conditions anywhere in the causal pathway (including as the underlying condition; presumed community-acquired: N=306; presumed hospital-acquired: N=149). The right panels show underlying causes of death when pneumonia was either an immediate or antecedent cause of death (presumed community-acquired pneumonia: N=226; presumed hospital-acquired pneumonia: N=135). CHAMPS=Child Health and Mortality Prevention Surveillance.

71 decedents with pneumonia-associated deaths had HIV, with HIV attributed in the causal pathway in 66 (92·9%) of these cases. 28 (39·4%) of 71 decedents with HIV had been on antiretroviral treatment; among the 71 decedents with HIV who had pneumonia-associated deaths, 15 (21·1%) had *Pneumocystis jirovecii* in the causal chain. Furthermore, 182 (40·0%) 455 decedents with pneumonia-associated deaths were malnourished, of whom 16 (8·8%) had HIV, 24 (13·2%) had congenital birth defects, four (2·2%) had neurological disorders, and two (1·1%) had tuberculosis as the concomitant underlying condition (figure 1). Malnutrition was attributed in the causal pathway of 128 (28·1%) of 455 pneumonia-associated deaths, including six decedents with HIV. Four presumed hospital-acquired pneumonia cases had tuberculosis in the causal chain, two each with miliary and extra-pulmonary tuberculosis (appendix p 14).

Of the 455 decedents with pneumonia anywhere in the causal pathway, 1535 organisms were detected on PCR test of lung specimens (appendix p 15). The DeCoDe panel attributed 561 (36·5%) of the 1535 detected organisms in the pathogenesis of pneumonia-associated deaths (appendix p 16). Causative pathogens were ascertained in 377 (82·9%) of 455 pneumonia-associated deaths, whereas no pathogen was ascribed in 78 (17·1%) of 455 cases. Among pneumonia-associated deaths with identifiable pathogens, more than one pathogen was attributed in 218 (57·8%) of 377 cases (table 2). The permutations of pathogens in pneumonia-associated deaths attributed to more than one organism included bacterial-bacterial (154 [70·6%] of 218), bacterial-viral (81 [37·2%] of 218), bacterial-fungal (26 [11·9%] of 218), fungal-viral (17 [7·8%] of 218), and viral-viral (16 [7·3%] of 218) combinations (appendix p 29).

**Table 2:** Pathogens identified as causing deaths due to pneumonia in the CHAMPS Network, stratified by age group and whether they were presumed to be community-acquired or hospital-acquired pneumonia, from Dec 16, 2016, to Dec 31, 2022

			**Community deaths or deaths occurring in <72 h of hospital admissio**n				**Deaths occurring ≥72 h after hospital admissio**n			
			All (N=306)	1–6 months (N=104)	6–12 months (N=66)	12–59 months (N=136)	All (N=149)	1–6 months (N=66)	6–12 months (N=28)	12–59 months (N=55)
Pathogen										
	Gram-negative bacteria		148 (48·4%)	53 (51·0%)	28 (42·4%)	67 (49·3%)	84 (56·4%)	43 (65·2%)	12 (42·9%)	29 (52·7%)
		*Klebsiella pneumoniae*	78 (25·5%)	31 (29·8%)	15 (22·7%)	32 (23·5%)	64 (43·0%)	32 (48·5%)	8 (28·6%)	24 (43·6%)
		Non-typeable *Haemophilus influenzae*	37 (12·1%)	9 (8·7%)	7 (10·6%)	21 (15·4%)	8 (5·4%)	2 (3·0%)	2 (7·1%)	4 (7·3%)
		*Acinetobacter baumanni*i	11 (3·6%)	8 (7·7%)	1 (1·5%)	2 (1·5%)	19 (12·8%)	12 (18·2%)	3 (10·7%)	4 (7·3%)
		*Pseudomonas aeruginosa*	10 (3·3%)	3 (2·9%)	1 (1·5%)	6 (4·4%)	15 (10·1%)	5 (7·6%)	3 (10·7%)	7 (12·7%)
		*Escherichia coli*	14 (4·6%)	5 (4·8%)	3 (4·5%)	6 (4·4%)	2 (1·3%)	2 (3·0%)	0	0
		*Haemophilus influenzae* Type A	14 (4·6%)	3 (2·9%)	2 (3·0%)	9 (6·6%)	2 (1·3%)	0	1 (3·6%)	1 (1·8%)
		*Moraxella catarrhalis*	12 (3·9%)	4 (3·8%)	3 (4·5%)	5 (3·7%)	1 (0·7%)	0	0	1 (1·8%)
		*Bordetella pertussis*	4 (1·3%)	4 (3·8%)	0	0	3 (2·0%)	3 (4·5%)	0	0
		*Haemophilus influenzae* Type B	3 (1·0%)	2 (1·9%)	1 (1·5%)	0	0	0	0	0
		*Klebsiella* spp*	2 (0·7%)	0	1 (1·5%)	1 (0·7%)	1 (0·7%)	0	1 (3·6%)	0
		*Salmonella* spp	3 (1·0%)	0	1 (1·5%)	2 (1·5%)	0	0	0	0
		Other†	6 (2·0%)	3 (2·9%)	1 (1·5%)	3 (2·2%)	2 (1·3%)	2 (3·0%)	0	0
Gram-positive bacteria			142 (46·4%)	43 (41·3%)	36 (54·5%)	63 (46·3%)	40 (26·8%)	18 (27·3%)	5 (17·9%)	17 (30·9%)
	*Streptococcus pneumoniae*		108 (35·3%)	28 (26·9%)	30 (45·5%)	50 (36·8%)	15 (10·1%)	5 (7·6%)	3 (10·7%)	7 (12·7%)
	*Staphylococcus aureus*		17 (5·6%)	7 (6·7%)	3 (4·5%)	7 (5·1%)	14 (9·4%)	9 (13·6%)	1 (3·6%)	4 (7·3%)
	*Streptococcus* spp		19 (6·2%)	7 (6·7%)	4 (6·1%)	8 (5·9%)	7 (4·7%)	2 (3·0%)	1 (3·6%)	4 (7·3%)
	*Streptococcus viridans*		1 (0·3%)	0	1 (1·5%)	0	3 (2·0%)	2 (3·0%)	0	1 (1·8%)
	*Enterococcus faecalis*		0	0	0	0	3 (2·0%)	1 (1·5%)	0	2 (3·6%)
	*Streptococcus agalactiae*		2 (0·7%)	1 (1·0%)	0	1 (0·7%)	1 (0·7%)	0	0	1 (1·8%)
	Other†		3 (1·0%)	3 (2·9%)	0	0	1 (0·7%)	0	0	1 (1·8%)
Virus			72 (23·5%)	29 (27·9%)	18 (27·3%)	25 (18·4%)	73 (49·0%)	38 (57·6%)	14 (50·0%)	21 (38·2%)
	Cytomegalovirus		28 (9·2%)	15 (14·4%)	7 (10·6%)	6 (4·4%)	26 (17·4%)	14 (21·2%)	6 (21·4%)	6 (10·9%)
	Respiratory syncytial virus		15 (4·9%)	8 (7·7%)	4 (6·1%)	3 (2·2%)	14 (9·4%)	9 (13·6%)	0	5 (9·1%)
	Adenovirus		6 (2·0%)	2 (1·9%)	1 (1·5%)	3 (2·2%)	19 (12·8%)	8 (12·1%)	5 (17·9%)	6 (10·9%)
	Influenza A		7 (2·3%)	0	2 (3·0%)	5 (3·7%)	3 (2·0%)	1 (1·5%)	1 (3·6%)	1 (1·8%)
	Parainfluenza virus type 3		2 (0·7%)	0	1 (1·5%)	1 (0·7%)	8 (5·4%)	5 (7·6%)	1 (3·6%)	2 (3·6%)
	Rhinovirus		4 (1·3%)	1 (1·0%)	1 (1·5%)	2 (1·5%)	6 (4·0%)	5 (7·6%)	0	1 (1·8%)
	Human metapneumovirus		4 (1·3%)	2 (1·9%)	1 (1·5%)	1 (0·7%)	3 (2·0%)	1 (1·5%)	0	2 (3·6%)
	Influenza B		4 (1·3%)	2 (1·9%)	1 (1·5%)	1 (0·7%)	2 (1·3%)	0	1 (3·6%)	1 (1·8%)
	Parainfluenza virus type 1		3 (1·0%)	0	2 (3·0%)	1 (0·7%)	2 (1·3%)	0	1 (3·6%)	1 (1·8%)
	Parainfluenza virus type 4		2 (0·7%)	0	1 (1·5%)	1 (0·7%)	1 (0·7%)	0	1 (3·6%)	0
	Other†		1 (0·3%)	1 (1·0%)	0	0	1 (0·7%)	1 (1·5%)	0	0
Fungus			24 (7·8%)	15 (14·4%)	2 (3·0%)	7 (5·1%)	11 (7·4%)	7 (10·6%)	3 (10·7%)	1 (1·8%)
	*Pneumocystis jiroveci*i		19 (6·2%)	13 (12·5%)	2 (3·0%)	4 (2·9%)	8 (5·4%)	6 (9·1%)	1 (3·6%)	1 (1·8%)
	*Candida albicans*		3 (1·0%)	0	0	3 (2·2%)	0	0	0	0
	*Candida* spp		2 (0·7%)	2 (1·9%)	0	0	1 (0·7%)	0	1 (3·6%)	0
	Other†		0	0	0	0	2 (1·3%)	1 (1·5%)	1 (3·6%)	0
No pathogen implicated			63 (20·6%)	21 (20·2%)	13 (19·7%)	29 (21·3%)	15 (10·1%)	1 (1·5%)	4 (14·3%)	10 (18·2%)
Number of cases with only one pathogen implicated			106 (34·6%)	31 (29·8%)	23 (34·8%)	52 (38·2%)	53 (35·6%)	23 (34·8%)	11 (39·3%)	19 (34·5%)
Number of cases with two pathogens implicated			71 (23·2%)	23 (22·1%)	16 (24·2%)	32 (23·5%)	44 (29·5%)	25 (37·9%)	8 (28·6%)	11 (20·0%)
Number of cases with three pathogens implicated			66 (21·6%)	29 (27·9%)	14 (21·2%)	23 (16·9%)	33 (22·1%)	14 (21·2%)	5 (17·9%)	14 (25·5%)
Number of cases with four or more pathogens implicated			0	0	0	0	4 (2·7%)	3 (4·5%)	0	1 (1·8%)
Median number of pathogens implicated per case‡			2 (1–3)	2 (1–3)	2 (1–3)	2 (1–2)	2 (1–3)	2 (1–3)	2 (1–2)	2 (1–3)

Data are n (%) or median (IQR). CHAMPS=Child Health and Mortality Prevention Surveillance. Deaths attributed to pneumonia that occurred in the community or within 72 h of hospital admission were presumed to be community-acquired pneumonia and those that occurred 72 h or more after hospital admission were presumed to be hospital-acquired pneumonia.

* Includes *Klebsiella species* other than *Klebsiella pneumoniae*.

† Other organisms reported in the appendix (p 16).

‡ Restricted to pneumonia-associated deaths with at least one pathogen implicated.

Of all the pneumonia-associated deaths, 104 (61·1%) of 170 in the early infancy group, 66 (70·2%) of 94 in the late infancy group, and 136 (71·2%) of 191 in the childhood age group were classified as presumed community-acquired pneumonia cases (table 2). 99 (32·4%) of 306 deaths from presumed community-acquired pneumonia occurred in the community. The leading bacterial pathogens in presumed community-acquired pneumonia deaths were *Streptococcus pneumoniae* (108 [35·3%] of 306), *Klebsiella pneumoniae* (78 [25·5%] of 306), non-typeable *Haemophilus influenzae* (37 [12·1%] of 306), and *Staphylococcus aureus* (17 [5·6%] of 306). *S pneumoniae* was a more common cause of presumed community-acquired pneumonia in late infancy (30 [45·5%] of 66) and childhood (50 [36·8%] of 136) than in early infancy (28 [26·9%] of 104). The prevalence of *K pneumoniae* as a cause of death due to presumed community-acquired pneumonia was similar in the early infancy (31 [29·8%] of 104), late infancy (15 [22·7%] of 66), and childhood (32 [23·5%] of 136) age groups. Overall, among the deaths due to presumed community-acquired pneumonia, the most prevalent viruses and fungi attributed in the pathogenesis were cytomegalovirus (28 [9·2%] of 306), respiratory syncytial virus (15 [4·9%] of 306), influenza A/B (11 [3·6%] of 306), and *P jirovecii* (19 [6·2%] of 306). In early infancy, cytomegalovirus was implicated in 15 (14·4%) of 104 deaths, respiratory syncytial virus was implicated in eight (7·7%) of 104 deaths, and *P jirovecii* was implicated in 13 (12·5%) of 104 deaths, all from presumed community-acquired pneumonia. In presumed community-acquired pneumonia cases with respiratory syncytial virus (n=15) and influenza A/B (n=11) attributed in the causal pathway, six (40·0%) of 15 cases of respiratory syncytial virus and seven (63·6%) of 11 cases of influenza A/B had bacterial co-infections. Furthermore, among the 137 presumed community-acquired pneumonia cases with more than one attributable pathogen, common co-pathogens combinations were *K pneumoniae* with *S pneumoniae* (28 [20·4%] of 137) and *S pneumoniae* with non-typeable *H influenzae* (23 [16·7%] of 137; table 2; appendix pp 17–18, 30).

Of all pneumonia-associated deaths, 66 (38·8%) of 170 in the early infancy group, 28 (29·8%) of 94 in the late infancy group, and 55 (28·8%) of 191 in the childhood age group were from presumed hospital-acquired pneumonia (Table 2, Table 3). The leading bacterial pathogens in deaths from presumed hospital-acquired pneumonia were *K pneumoniae* (64 [43·0%] of 149), *Acinetobacter baumannii* (19 [12·8%] of 149), *S pneumoniae* (15 [10·1%] of 149), and *Pseudomonas aeruginosa* (15 [10·1%] of 149). *K pneumoniae* was more commonly attributed as a cause of death from presumed hospital-acquired pneumonia in early infancy (32 [48·5%] of 66) and childhood (24 [43·6%] of 55) than in late infancy (eight [28·6%] of 28). The most prevalent viruses among deaths due to presumed hospital-acquired pneumonia were cytomegalovirus (26 [17·4%] of 149), adenovirus (19 [12·8%] of 149), and respiratory syncytial virus (14 [9·4%] of 149). *P jirovecii* was implicated in eight (5·4%) of 149 deaths associated with presumed hospital-acquired pneumonia, six (75·0%) of which occurred in the early infancy age group (table 2; appendix pp 17–18). The sensitivity analysis in which we defined deaths due to presumed hospital-acquired pneumonia as those occurring at least 120 h after hospital admission (n=111) yielded similar findings compared with when we defined deaths from presumed hospital-acquired pneumonia as those occurring at least 72 h after hospital admission (table 2; appendix pp 19–20).

**Table 3:** Pathogens identified as causing deaths due to pneumonia in the CHAMPS Network, for all pneumonia deaths and stratified by whether the pneumonia was the underlying or immediate or antecedent cause of death, and whether it was presumed to be community-acquired or hospital-acquired pneumonia, from Dec 16, 2016, to Dec 31, 2022

		**Community deaths or deaths occurring in <72 h of hospital admission**			**Deaths occurring ≥72 h after hospital admission**		
		All pneumonia deaths (N=306)*	Pneumonia was underlying cause of death (N=88)	Pneumonia was immediate or antecedent cause of death (N=226)	All pneumonia deaths (N=149)*	Pneumonia was underlying cause of death (N=17)	Pneumonia was immediate or antecedent cause of death (N=135)
Gram-negative bacteria		148 (48·4%)	37 (42·0%)	113 (50·0%)	84 (56·4%)	8 (47·1%)	77 (57·0%)
	Klebsiella pneumoniae	78 (25·5%)	15 (17·0%)	63 (27·9%)	64 (43·0%)	5 (29·4%)	59 (43·7%)
	Non-typeable *Haemophilus influenzae*	37 (12·1%)	7 (8·0%)	30 (13·3%)	8 (5·4%)	2 (11·8%)	6 (4·4%)
	*Acinetobacter baumannii*	11 (3·6%)	1 (1·1%)	10 (4·4%)	19 (12·8%)	1 (5·9%)	18 (13·3%)
	*Pseudomonas aeruginosa*	10 (3·3%)	1 (1·1%)	9 (4·0%)	15 (10·1%)	0	15 (11·1%)
	*Escherichia coli*	14 (4·6%)	1 (1·1%)	13 (5·8%)	2 (1·3%)	0	2 (1·5%)
	*Haemophilus influenzae* Type A	14 (4·6%)	7 (8·0%)	7 (3·1%)	2 (1·3%)	0	2 (1·5%)
	*Moraxella catarrhalis*	12 (3·9%)	3 (3·4%)	9 (4·0%)	1 (0·7%)	0	1 (0·7%)
	*Bordetella pertussis*	4 (1·3%)	4 (4·5%)	0	3 (2·0%)	1 (5·9%)	3 (2·2%)
	*Haemophilus influenzae* Type B	3 (1·0%)	2 (2·3%)	1 (0·4%)	0	0	0
	*Klebsiella* spp†	2 (0·7%)	1 (1·1%)	1 (0·4%)	1 (0·7%)	0	1 (0·7%)
	*Salmonella* spp	3 (1·0%)	0	3 (1·3%)	0	0	0
	Other‡	6 (2·0%)	3 (3·4%)	4 (1·8%)	2 (1·3%)	0	2 (1·5%)
Gram-positive bacteria		142 (46·4%)	35 (39·8%)	108 (47·8%)	40 (26·8%)	1 (5·9%)	39 (28·9%)
	*Streptococcus pneumoniae*	108 (35·3%)	27 (30·7%)	81 (35·8%)	15 (10·1%)	1 (5·9%)	14 (10·4%)
	*Staphylococcus aureus*	17 (5·6%)	3 (3·4%)	15 (6·6%)	14 (9·4%)	0	14 (10·4%)
	*Streptococcus* spp	19 (6·2%)	5 (5·7%)	14 (6·2%)	7 (4·7%)	0	7 (5·2%)
	*Streptococcus viridans*	1 (0·3%)	0	1 (0·4%)	3 (2·0%)	0	3 (2·2%)
	*Enterococcus faecalis*	0	0	0	3 (2·0%)	0	3 (2·2%)
	*Streptococcus agalactiae*	2 (0·7%)	1 (1·1%)	1 (0·4%)	1 (0·7%)	0	1 (0·7%)
	Other‡	3 (1·0%)	0	3 (1·3%)	1 (0·7%)	0	1 (0·7%)
Virus		72 (23·5%)	19 (21·6%)	55 (24·3%)	73 (49·0%)	10 (58·8%)	63 (46·7%)
	Cytomegalovirus	28 (9·2%)	4 (4·5%)	24 (10·6%)	26 (17·4%)	2 (11·8%)	24 (17·8%)
	Respiratory syncytial virus	15 (4·9%)	7 (8·0%)	8 (3·5%)	14 (9·4%)	3 (17·6%)	11 (8·1%)
	Adenovirus	6 (2·0%)	0	6 (2·7%)	19 (12·8%)	5 (29·4%)	14 (10·4%)
	Influenza A	7 (2·3%)	2 (2·3%)	5 (2·2%)	3 (2·0%)	0	3 (2·2%)
	Parainfluenza virus type 3	2 (0·7%)	0	2 (0·9%)	8 (5·4%)	0	8 (5·9%)
	Rhinovirus	4 (1·3%)	3 (3·4%)	1 (0·4%)	6 (4·0%)	0	6 (4·4%)
	Human metapneumovirus	4 (1·3%)	3 (3·4%)	1 (0·4%)	3 (2·0%)	0	3 (2·2%)
	Influenza B	4 (1·3%)	1 (1·1%)	4 (1·8%)	2 (1·3%)	1 (5·9%)	1 (0·7%)
	Parainfluenza virus type 1	3 (1·0%)	1 (1·1%)	2 (0·9%)	2 (1·3%)	0	2 (1·5%)
	Parainfluenza virus type 4	2 (0·7%)	0	2 (0·9%)	1 (0·7%)	0	1 (0·7%)
	Other‡	1 (0·3%)	0	2 (0·9%)	1 (0·7%)	0	1 (0·7%)
Fungus		24 (7·8%)	4 (4·5%)	20 (8·8%)	11 (7·4%)	2 (11·8%)	9 (6·7%)
	*Pneumocystis jirovecii*	19 (6·2%)	4 (4·5%)	15 (6·6%)	8 (5·4%)	2 (11·8%)	6 (4·4%)
	Candida albicans	3 (1·0%)	0	3 (1·3%)	0	0	0
	*Candida* spp	2 (0·7%)	0	2 (0·9%)	1 (0·7%)	0	1 (0·7%)
	Other‡	0	0	0	2 (1·3%)	0	2 (1·5%)
No pathogen implicated		63 (20·6%)	26 (29·5%)	37 (16·4%)	15 (10·1%)	1 (5·9%)	14 (10·4%)

Data are n (%) or median (IQR). CHAMPS=Child Health and Mortality Prevention Surveillance. Deaths attributed to pneumonia that occurred in the community or within 72 h of hospital admission were presumed to be community-acquired pneumonia and those that occurred 72 h or more after hospital admission were presumed to be hospital-acquired pneumonia.

* Several decedents had pneumonia as an underlying and immediate or antecedent cause of death, so the number of underlying plus immediate or antecedent count for pneumonia episodes exceeds the total number of decedents.

† Includes *Klebsiella* species other than *Klebsiella pneumoniae*.

‡ See the appendix (pp 17–18) for details of other pathogens.

**Figure 2:**
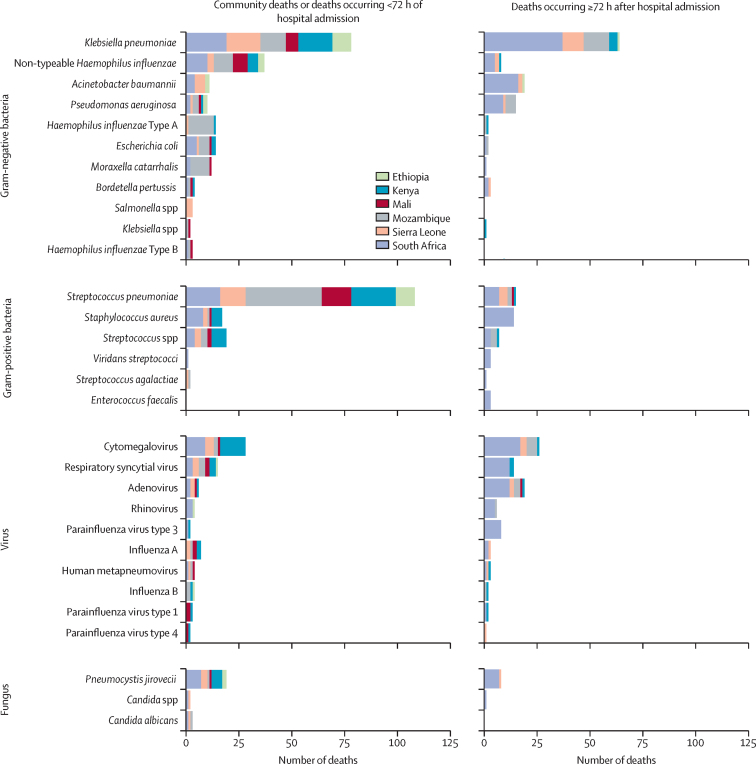
Pathogens attributed to deaths from pneumonia, based on CHAMPS Network data for Dec 16, 2016, to Dec 31, 2022 Pathogens attributed to deaths from pneumonia in children aged 1–59 months enrolled in the CHAMPS Network, by CHAMPS site and by whether the pneumonia death occurred in the community or less than 72 h of hospital admission or whether it occurred 72 h or more after hospital admission. Pathogens implicated in at least three deaths were included. CHAMPS=Child Health and Mortality Prevention Surveillance.

In the analysis stratified by country for deaths associated with presumed community-acquired pneumonia, *S pneumoniae* was the most prevalent pathogen in Kenya (14 [32·3%] of 66), Mozambique (14 [52·3%] of 68), Mali (14 [50·0%] of 28), and Ethiopia (nine [47·4%] of 19; figure 2). *K pneumoniae* was the second most common pathogen among deaths from presumed community-acquired pneumonia across five of the sub-Saharan African sites, ranging from 12 (17·6%) of 68 in Mozambique to nine (47·4%) of 19 in Ethiopia, while it was the most common cause in South Africa (19 [31·1%] of 61; figure 2; appendix p 21).

When analysing all pneumonia-associated deaths, South Africa had the highest proportion of cases attributed to viruses (69 [45·7%] of 151) and fungi (19 [12·6%] of 151). The proportion of overall pneumonia deaths attributed to respiratory syncytial virus was also highest in South Africa (15 [9·9%] of 151). Overall pneumonia deaths attributed to cytomegalovirus were common in Kenya (13 [16·7%] of 78) and South Africa (26 [17·2%] of 151; appendix pp 22–23). The majority of pneumonia deaths attributed to *A baumannii* (20 [66·7%] of 30, including 16 from presumed hospital-acquired pneumonia) and *S aureus* (22 [71·0%] of 31, including 14 from presumed hospital-acquired pneumonia) were from South Africa (appendix pp 22–24).

Overall, 52 (96·3%) of 54 pneumonia deaths attributed to cytomegalovirus were graded as level 1 evidence, and two (3·7%) of 54 were graded as level 2 evidence. Among the pneumonia-associated deaths attributed to cytomegalovirus, 47 (87·0%) of 54 had histological evidence of changes in the lung, including intranuclear inclusion bodies in 31 (57·4%) cases and positive immunohistochemistry in 40 (74·1%). Ten of the 43 cases with cytomegalovirus lung histopathology findings also had cytomegalovirus-associated histopathological findings in the liver. The median cycle threshold (Ct) on TaqMan Array Cards for cytomegalovirus on lung samples (n=51) where it was attributed as a pathogen was lower (median Ct 25·9) than in cases where it was detected (n=230) but not attributed in the causal pathway (median Ct 31·2), and similarly so on nasopharyngeal samples (appendix p 25). The majority of deaths from cytomegalovirus-associated pneumonia were in children with HIV (28 [51·9%] of 54) and malnutrition (17 [31·5%] of 54) as concurrent conditions. Furthermore, 38 (70·4%) of 54 cytomegalovirus-associated deaths had other co-infecting pathogens implicated in the same pneumonia episode attributed to cytomegalovirus, including *P jirovecii* (n=12), *S pneumoniae* (n=11), *K pneumoniae* (n=9), adenovirus (n=4), *A baumannii* (n=3), *Bordetella pertussis* (n=3), and respiratory syncytial virus (n=3).

The DeCoDe panel determined that 373 (82·2%) of 455 pneumonia-associated deaths could have been preventable, including through improved clinical management (191 [55·0%] of 347), health education (134 [38·6%] of 347), health-seeking behaviour (113 [32·6%] of 347), infection prevention and control in health facilities (97 [28·0%] of 347), and nutritional support (94 [27·1%] of 347; appendix pp 31–32).

## Discussion

The CHAMPS Network surveillance provides in-depth insight into the role of pneumonia in the causal pathway of deaths and the causative pathogens of fatal pneumonia in children. When considering pneumonia at any stage of the causal pathway to death, rather than only as an underlying cause as done in modelling studies on causes of death attribution,^15^, ^16^ pneumonia played a role in 40·6% of all decedents in our study. Pneumonia was identified as the underlying cause of death in only 105 (9·4%) of 1120 decedents aged 1–59 months in our study, compared with 22% in previously published modelling studies on causes of childhood deaths.^15^, ^16^ Additionally, we provide granular information on the pathogens contributing to pneumonia-associated deaths. Notably, one or more pathogens was attributed in the pathogenesis of 79·4% of presumed community-acquired pneumonia cases.

Although many studies have explored the epidemiological and clinical characteristics of pneumonia, few have done so by investigating the causative pathogens through direct lung tissue sampling. Ante-mortem diagnostic testing of pneumonia in children typically involves indirect measures such as culture or PCR of nasopharyngeal swabs, blood samples, or induced sputum, with lung sampling rarely undertaken. Blood cultures, which are commonly used to diagnose bacterial pneumonia in children, have limited sensitivity (around 5%).^17^ The Pneumonia Etiology Research for Child Health (PERCH) study reported positive blood cultures in only 3·2% of pneumonia cases with radiologically confirmed pneumonia.^18^ Our study followed a comprehensive approach, utilising immunohistochemistry assays and pathology examination of lung tissue, alongside NAAT analysis for multiple organisms on blood, lung, and nasopharyngeal swab samples.

Our study highlights that despite widespread use of the pneumococcal conjugate vaccine (PCV) across all participating countries, *S pneumoniae* remained the leading cause (35·3%) of presumed community-acquired pneumonia, emphasising the need for additional interventions targeting *S pneumoniae*. Our data suggest that *S pneumoniae* remains a leading vaccine-preventable cause in children, with modelled estimates suggesting it caused 318 000 childhood deaths globally in 2015.^19^ Considering the substantial reduction in circulation of serotypes included in PCV due to childhood immunisation,^20^ it is likely that most of the deaths due to pneumococcal pneumonia were caused by serotypes not included in current 10–13-valency PCV formulations. Incomplete PCV vaccination could also have contributed to the ongoing burden of deaths due to pneumococcal-associated pneumonia. Unfortunately, vaccine records were not available to quantify the immunisation status of the children included in the CHAMPS Network programme. Serotype characterisation of the *S pneumoniae* isolates from the study is underway.

Our study also highlighted the hitherto under-appreciated role of *K pneumoniae* as the second most important pathogen (25·5%) in presumed community-acquired pneumonia and the most common pathogen (43·0%) in presumed hospital-acquired pneumonia. *K pneumoniae* is a prevalent pathogen in low-income, middle-income, and high-income countries, spreading rapidly within hospitals.^21^ *K pneumoniae* is estimated to account for 3–5% of community-acquired pneumonia cases across all age groups in high-income countries compared with 15% in low-income and middle-income countries.^22^ Our findings emphasise the need for diagnostics methods that can quickly identify cases of pneumonia due to *K pneumoniae* and the need for tailored empirical antibiotic therapy in cases of severe pneumonia in low-income and middle-income settings. The current WHO recommendation for first-line empirical treatment for childhood community-acquired pneumonia is amoxicillin or amoxicillin–clavulanic acid, which would be inadequate in the treatment of *K pneumoniae*, and could have contributed to the prominent role of *K pneumoniae* even in deaths due to presumed community-acquired pneumonia in our study.

The presence of Gram-negative pathogens in post-mortem samples raises concerns about possible contamination or overgrowth in post-mortem samples. Nevertheless, the DeCoDe panel considered pathology findings when attributing organisms to the causal chain of death, only if there was strong histopathology evidence or clinical presentation supporting the diagnosis.^7^ The two most common Gram-negative pathogens identified were *K pneumoniae* and non-typeable *H influenzae,* which have also been associated with high morbidity and mortality in children younger than 5 years in other studies.^16^, ^23^, ^24^ Although our study also identified *A baumannii* as an important cause of presumed hospital-acquired pneumonia in South Africa, we recognise the need for additional data to generalise this finding to the entire country or other countries.

Respiratory syncytial virus has been attributed as the most common cause of severe and very severe community-acquired pneumonia in children admitted to hospital (31%) in the PERCH study involving countries in sub-Saharan Africa and South Asia.^25^ The PERCH study mainly involved ante-mortem blood culture and NAATs of upper respiratory tract samples to ascertain the causes of community-acquired pneumonia; *S pneumoniae* was attributed as the causative pathogen in 4·6% of severe pneumonia cases and 9·7% of very severe pneumonia cases. The paucity of lung sampling could have contributed to an underestimation of the role of *S pneumoniae* and other bacteria as a causative organism in PERCH.^26^ Furthermore, PERCH did not evaluate for *K pneumoniae* as a potential pathogen in the pneumonia cases, due to concerns related to the NAAT assay used in the study. Consequently, differences in the spectrum of pathogens attributed to severe and very severe pneumonia cases in PERCH compared with our findings highlight the need for post-mortem direct sampling of the lung to adequately characterise the contribution of different pathogens in the pathogenesis of fatal pneumonia. Moreover, pathogen-specific differences in case fatality risk, such as the lower risk for respiratory syncytial virus compared with pneumococcal pneumonia,^25^ could contribute to differences in the proportionality of cases attributed to different pathogens in PERCH compared with the CHAMPS Network.

PERCH did not identify cytomegalovirus as a major cause of pneumonia-related deaths, showing similar prevalence of cytomegalovirus in upper airway swabs of children admitted to hospital with pneumonia and healthy children in the control group.^18^ By contrast, our study provides histopathology evidence from lung tissue confirming the role of cytomegalovirus in 9·2% of deaths from presumed community-acquired pneumonia and 17·4% of deaths from presumed hospital-acquired pneumonia. Notably, approximately a third of cytomegalovirus-attributed deaths from pneumonia occurred in malnourished children without HIV. Comorbidities including malnutrition, anaemia, and HIV can influence pneumonia severity and lethality by modulating the impact of aetiological agents.^27^ Another finding in our study is the high prevalence of *P jirovecii,* the most common fungus identified in pneumonia-related deaths in the CHAMPS Network, particularly among children with HIV (21·1%).^16^, ^28^

Malnutrition, which is not included as an underlying cause of death in global childhood mortality estimates,^1^ was a dominant comorbidity in the pneumonia-related deaths in our study. Severe malnutrition compromises cell-mediated and humoral immune responses, rendering children highly susceptible to infectious diseases, including pneumonia.^29^

The CHAMPS Network aims to provide information on how childhood deaths can be reduced, and the DeCoDe panellists provided clinical and public health recommendations on some factors that could have mitigated the deaths.^30^ Our findings that more than two-thirds of deaths from pneumonia were potentially preventable underscore the potential for current interventions to reduce pneumonia-related mortality rates. Further investigation is required to identify specific clinical management failures contributing to these deaths.

Limitations of the CHAMPS programme include the fact that prompt identification of deaths for timely sample collection can lead to an over-representation of presumed hospital-acquired pneumonia, potentially skewing the proportional distribution of deaths from presumed community-acquired pneumonia and presumed hospital-acquired pneumonia. Furthermore, the ease of identifying deaths in a health facility compared with in the community could introduce selection bias, favouring the inclusion of children from families with access to health care. Nevertheless, the diversity of our African study sites enhances the generalisability of our findings to sub-Saharan Africa, where 75% of all under-5 deaths occurred in 2019.^31^ The sites in sub-Saharan Africa showed some heterogeneity, which was expected given their diverse nature. Currently, CHAMPS has scarce data from South Asian countries, with only two pneumonia cases from Bangladesh included in this report. Additional study sites will be launched in Pakistan in 2024. A further limitation of CHAMPS is the criteria for stratification of presumed community-acquired pneumonia and presumed hospital-acquired pneumonia. Our definition of presumed community-acquired pneumonia and presumed hospital-acquired pneumonia is subject to misclassification in either direction, considering the potential for variations in clinical deterioration patterns and uncertainty in the timing of health-care-associated infections or previous exposure to health-care settings in decedents. Misclassification of cases as presumed hospital-acquired pneumonia might especially be pertinent for organisms such as respiratory viruses, which could be insidious in the clinical course, and could have ongoing shedding and identification beyond 72 h after admission.

In summary, our study identified pneumonia as a major cause of mortality in low-income and middle-income countries with high under-5 mortality rates. By using MITS, we identified specific pathogens associated with pneumonia-attributed deaths, often involving multiple aetiological agents. Our findings suggest that *K pneumoniae* and cytomegalovirus might have a greater role in deaths from pneumonia than previously recognised. Despite widespread PCV use across all sites, *S pneumoniae* remains a major contributor to childhood deaths from pneumonia, particularly in sub-Saharan Africa. These findings underscore the importance of enhancing pneumonia prevention efforts, including improved vaccine coverage, awareness, and more effective treatment strategies, such as enhanced clinical management.

## Data sharing

Summarised data are publicly available through the CHAMPS website. Requests for further detailed data for research and evaluation purposes can be made at: https://champshealth.org/data/.

## Declaration of interests

CGW received honoraria from the University of St Andrews for speaking to alumni about CHAMPS and global health work. SAM has received grants from the Bill & Melinda Gates Foundation, GSK, Pfizer, Minervax, Novavax, Merck, Providence, Gritstone, and ImmunityBio. SAM has received honoraria from GSK for lecturing. GR has received grants from Fleming Fund Kenya Country, Deutsche Forschungsgemeinschaft, and bioMerieux. SA and JAGS have received support for attending meetings or travels, or both, for WHO, Bill & Melinda Gates Foundation (SA) and the International Society of Pneumonia & Pneumococcal Diseases (JAGS). CGW, JAGS, and SAM report serving on data safety monitoring boards for Safety Platform for Emergency VACcines (SPEAC; CGW), PATH (SAM), Centre for the AIDS Programme of Research in South Africa (CAPRISA; SAM), MRC The Gambia (JAGS) and ILiAD Biotechnologies (JAGS). All other authors declare no competing interests.
