# Supplementary material for: Post-mortem investigation of deaths due to pneumonia in children aged 1–59 months in sub-Saharan Africa and South Asia from 2016 to 2022: an observational study
Source: Lancet Child Adolesc Health. 2024 Mar;8(3):201–13. doi: 10.1016/S2352-4642(23)00328-0 (PMC10864189; doi:10.1016/S2352-4642(23)00328-0)
Supplement: Supplementary appendix [file mmc1.pdf]

THE LANCET
Child & Adolescent Health

Supplementary appendix

This appendix formed part of the original submission and has been peer reviewed. We post it as supplied by the authors.

Supplement to: Mahtab S, Blau DM, Madewell ZJ, et al. Post-mortem investigation of deaths due to pneumonia in children aged 1–59 months in sub-Saharan Africa and South Asia from 2016 to 2022: an observational study. *Lancet Child Adolesc Health* 2024; published online Jan 25. https://doi.org/10.1016/S2352-4642(23)00328-0.

Appendix to "Post-mortem Investigation of Pneumonia Deaths in Children 1-59 Months in sub-Saharan Africa and South Asia countries: An observational study from 2016 to 2022"

Sana Mahtab, Dianna M. Blau, Zachary J. Madewell, on behalf of the CHAMPS consortium, Ikechukwu Ogbuanu, Julius Ojulong, Sandra Lako, Hailemariam Legesse, Joseph S. Bangura, Quique Bassat, Inacio Mandomando, Elisio Xerinda, Fabiola Fernandes, Rosauro Varo, Samba O. Sow, Karen L. Kotloff, Milagritos D. Tapia, Adama Mamby Keita, Diakaridia Sidibe, Dickens Onyango, Victor Akelo, Dickson Gethi, Jennifer K. Verani, Gunturu Revathi, J. Anthony G. Scott, Nega Assefa, Lola Madrid, Hiwot Bizuayehu, Tseyon Tesfaye Tirfe, Shams El Arifeen, Emily S. Gurley, Kazi Munisul Islam, Muntasir Alam, Mohammad Zahid Hossain, Ziyaad Dangor, Vicky L. Baillie, Martin Hale, Portia Mutevedzi, Robert F. Breiman, Cynthia G. Whitney, Shabir A. Madhi.

Table of Contents for Appendix

Contents	Page
**Supplemental Table S1.** Pathogen Targets (N = 116) Validated for Use on Child Health and Mortality Prevention Surveillance TaqMan Array Cards.	2-3
**Supplemental Table S2**. CHAMPS diagnosis standards for pneumonia and for pneumococcal pneumonia, an example of pneumonia due to a specific pathogen.	4-10
**Supplemental Table S3.** Causes of death in the causal chain for all 1120 deaths including those without pneumonia, CHAMPS Network, Africa and South Asia, December 16th 2016 to December 31st, 2022.	11
**Supplemental Table S4**. Ethnicities[*] of deceased children aged 1-59 months by whether pneumonia was attributed as a cause in the pathway leading to death (N = 1120), CHAMPS Network, Africa and South Asia, December 16th, 2016, to December 31st, 2022.	12
**Supplemental Table S5.** Characteristics of deaths attributed to pneumonia, by CHAMPS site.	13
**Supplemental Table S6:** Other conditions in the causal pathway to death when pneumonia is one of the conditions in the causal pathway stratified by age group and by whether the pneumonia death 1) occurred in the community or with fewer than 72 hours in the hospital or 2) occurred 72 or more hours after hospital admission, CHAMPS Network, December 16th 2016 to December 31st, 2022.	14
**Supplemental Table S7.** Pathogens in the causal chain for all 1120 deaths including those without pneumonia, CHAMPS Network, Africa and South Asia, December 16th 2016 to December 31st, 2022.	15
**Supplemental Table S8.** Pathogens identified as causing pneumonia deaths by age group, CHAMPS Network, December 16th 2016 to December 31st, 2022.	16
**Supplemental Table S9.** Pathogens identified as causing pneumonia deaths, for all pneumonia deaths and stratified by whether the pneumonia was the underlying, immediate, or antecedent cause of death and by whether the pneumonia death 1) occurred in the community or with fewer than 72 hours in the hospital or 2) 72 or more hours after hospital admission, CHAMPS Network, December 16th 2016 to December 31st, 2022.	17-18
**Supplemental Table S10.** Pathogens identified as causing pneumonia deaths stratified by age group and by whether the pneumonia death 1) occurred in the community or with fewer than 120 hours in the hospital or 2) occurred 120 or more hours after hospital admission[a], CHAMPS Network, December 16th 2016 to December 31st, 2022.	19-20
**Supplemental Table 11**: CHAMPS site specific analysis of pathogens attributed to causing pneumonia deaths which occurred in the community or within 72 hours of admission; CHAMPS Network, December 16th 2016 to December 31st, 2022	21
**Supplemental Table S12**: CHAMPS site specific analysis of pathogens attributed to causing overall pneumonia associated deaths; CHAMPS Network, December 16th 2016 to December 31st, 2022.	22-23
**Supplemental Table S13**: CHAMPS site specific analysis of pathogens attributed to causing pneumonia deaths which occurred 72 hours or later after hospital admission; CHAMPS Network, December 16th 2016 to December 31st, 2022.	24
**Supplemental Table S14:** Cycle threshold values for cytomegalovirus (CMV) on the TacMan Array Card nucleic acid amplification assay in children who tested positive for CMV, stratified by whether or not CMV was attributed in the etiology of pneumonia-associated death or not implicated in the causal pathway.	25
**Supplemental Figure 1.** Inclusion and exclusion criteria for minimally invasive tissue sampling (MITS) and non-MITS enrollment (ref *Clin Infect Dis*, Volume 69, Issue Supplement_4, 15 October 2019, Pages S262–S273.	26
**Supplemental Figure S2.** Flowchart of enrolled under-five infant and child deaths from CHAMPS sites from 2016 to 2022 that had minimally invasive tissue samples (MITS) and consent only for verbal autopsy and clinical abstraction (Non-MITS) and included in the analysis.	27
**Supplemental Figure 3**: Country-specific, age-group stratified childhood deaths with (Pneumonia+) or without (Pneumonia-) attributed in the causal pathway to death.	28
**Supplemental Figure 4**: Proportion of deaths determined to have died from a viral infection, bacterial infection, fungal infection, or co-infection for all pneumonia deaths and by whether the pneumonia death 1) occurred in the community or with fewer than 72 hours in the hospital or 2) or 72 or more hours after hospital admission, CHAMPS Network, December 16th 2016 to December 31st, 2022.	29
**Supplemental Figure 5.** Frequency of co-infections for each pathogen pair attributed to pneumonia, CHAMPS Network, December 16th 2016 to December 31st, 2022. Panel A includes deaths that occurred in the community or with fewer than 72 hours in the hospital (N=306); Panel B includes deaths which occurred 72 or more hours after hospital admission (N=149).	30
**Supplemental Figure 6:** Expert (DeCoDe) panel determination of whether pneumonia deaths were preventable (Figure 6a) and recommended improvements that could prevent such deaths (Figure 6b).	31-32
**Supplemental Results**	33
**CHAMPS Consortium**	34-35

Supplemental Table S1. Pathogen Targets (N = 116) Validated for Use on Child Health and Mortality Prevention Surveillance TaqMan Array Cards.

Pathogen	Target
**Bacteria:**	
Acinetobacter baumannii	R, B2
Aeromonas spp	E
Bartonella spp	B2
Bordetella parapertussis/B. bronchiseptica (pIS1001)	R
Bordetella pertussis/B. holmesii (IS481)	R
Brucella spp	B2
Burkholderia pseudomallei	R, B2
Campylobacter coli	E
Campylobacter jejuni	E
Chlamydia pneumoniae	R
Chlamydia trachomatis	R
Clostridioides difficile, nontoxigenic	E
C. difficile toxin A tcdA	E
C. difficile toxin B tcdB	E
Corynebacterium diphtheria	R
Corynebacterium pseudotuberculosis/C. ulcerans	R
C. diphtheriae/C. pseudotuberculosis/ C. ulcerans diphtheria toxin tox	R
Enterococcus faecalis	B2, E
Enterococcus faecium	B2, E
Enteroinvasive E. coli/Shigella spp ipaH	E
Enteropathogenic E. coli bfpA	E
Enteropathogenic E. coli eae	E
Enteroaggregative E. coli aatA	E
Enteroaggregative E. coli/Shigella spp ipaH	E
Enterotoxigenic E. coli heat-labile toxin eltA	E
Enterotoxigenic E. coli heat-stable toxin STh estA	E
Enterotoxigenic E. coli heat-stable toxin STp estA	E
Haemophilus influenzae	R, B1
H. influenzae type ba	R, B1
Klebsiella pneumoniae	R, B1
Leptospira	B2
Moraxella catarrhalis	R, B2
Mycobacterium tuberculosis	R, B2, E
Neisseria gonorrhoeae	B2
Neisseria meningitidis	B1
Orientia tsutsugamushi	B1
Rickettsia spp	B1
Salmonella enterica Paratyphi A	B1
S. enterica Typhi	B1
S. enterica/bongori	B1, E
Shiga toxin stx1	E
Shiga toxin stx2	E
Staphylococcus aureus	R, B1
Streptococcus agalactiae (GBS)	R, B1
Streptococcus pneumoniae	R, B1
Streptococcus pyogenes (GAS)	R, B1
Streptococcus suis	B2
Treponema pallidum	B1
Ureaplasma urealyticum/parvum	B2
Vibrio cholerae cholera toxin ctxA	E
**Viruses:**	
Adenovirus	R, B1, E
Adenovirus serotype 40/41	E
Astrovirus	E
Chikungunya virus	B1
Crimean-Congo hemorrhagic fever virus	B2
Cytomegalovirus	B2
Dengue virus	B1
Enterovirus	R, B1, E
Hepatitis E virus	B2
Human coronavirus 229E	R
Human coronavirus NL63	R
Human coronavirus OC43	R
Human coronavirus HKU1	R
Human herpesvirus 1	B2

Human herpesvirus 2	B2
Human metapneumovirus	R
Human parainfluenza virus 1	R
Human parainfluenza virus 2	R
Human parainfluenza virus 3	R
Human parainfluenza virus 4	R
Human parechovirus	B1
Influenza A virus	R
Influenza B virus	R
Japanese encephalitis virus	B2
Lassa fever virusc	B2
Lassa fever virus lineage 1/2/4	B2
MERS coronavirus N	R
MERS coronavirus upE	R
Measles virus	R, B2
Norovirus genogroup GI	E
Norovirus genogroup GII	E
Parvovirus B19	B2
Respiratory syncytial virus	R
Rhinovirus	R
Rift Valley fever virus	B2
Rotavirus A	E
Rotavirus B	E
Rotavirus C	E
Rotavirus, nontypeable	E
Rubella virus	R, B1
Sapovirus I/II/IV	E
Sapovirus V	E
Varicella zoster virus	R, B2
West Nile virus	B2
Yellow fever virus	B2
Zika virus	B1
**Parasites:**	
Ascaris lumbricoides	E
Cryptosporidium parvum	E
Entamoeba histolytica	E
Giardia intestinalis (lamblia)	E
Plasmodium falciparum	B1
Plasmodium vivax	B1
Toxoplasma gondii	B1
Trichuris trichiura	E
**Fungi:**	
Candida albicans	B2
Cryptococcus neoformans/gattii	B2
Pneumocystis jirovecii	R

Abbreviations: B1, blood/cerebrospinal fluid tier 1; B2, blood/CSF tier 2; E, enteric; GAS, group A Streptococcus; GBS, group B Streptococcus; MERS, Middle East respiratory syndrome; R, respiratory/lung.

Supplemental Table S2. CHAMPS diagnosis standards for pneumonia and for pneumococcal pneumonia, an example of pneumonia due to a specific pathogen. (REF https://champshealth.org/wp-content/uploads/2021/01/CHAMPS-Diagnosis-Standards.pdf)

Pneumonia
ICD-10 Codes: J18 (unspecified organism)

For pneumonia due to a specific pathogen, please reference the DS that follow. For aspiration pneumonia, please see J69.0.

Level 1

EITHER Strong histological evidence of pyogenic pneumonia in lung tissue

OR
One of the following laboratory or imaging findings
- Moderate histological evidence of pneumonia in lung tissue
- New infiltrate or pleural effusion on chest radiograph

AND TWO or more of the following clinical signs documented in the medical record:
- Tachypnea (Per WHO Clinical Case Definitions defined as respiratory rate >60/minute in 0-2 months, >50/minute for infants 2-12 months, >40 in children 12 months -5 years)
- Respiratory distress as chest indrawing, grunting or nasal flaring
- Abnormal breath sounds (i.e. decreased breath sounds, crackles, crepitations)
- Hypoxia, cyanosis or desaturations (oxygen saturation <95%)
- Fever >38.0 or hypothermia <36.0

Level 2

One of the following:
- No laboratory or imaging data available and ALL of the following documented in the medical record: fever or hypothermia, hypoxia or abnormal breath sounds, and tachypnea or respiratory distress.
- One of the laboratory or imaging findings above with TWO or more of clinical signs of pneumonia above reported by verbal autopsy (difficulty breathing, fast breathing or breathlessness, lower chest wall/ribs being pulled in or grunting, or fever).

Level 3

Acute febrile illness or hypothermia with tachypnea, respiratory distress, abnormal breath sounds, hypoxia or cyanosis documented in the medical record or reported by verbal autopsy, but not meeting the criteria for Level 1 or Level 2 diagnosis above OR laboratory evidence of pneumonia but not meeting the criteria for Level 1 or Level 2 diagnosis above.

Pneumonia due to *Streptococcus pneumoniae*
ICD-10 Code: J13

Level 1

One of the following:
- Strong histological evidence of pneumonia and detection of *S. pneumoniae* in lung tissue
- Moderate histological evidence of pneumonia and detection of *S. pneumoniae* in lung tissue with TWO of the clinical criteria for diagnosis of pneumonia as above, documented in medical record or reported by verbal autopsy.
- Pneumonia meeting TWO of the clinical criteria for diagnosis of pneumonia as above, as documented in the medical record, with one of the following:
  - Isolation of *S. pneumoniae* from blood culture with inadequate postmortem lung tissue
  - Detection of *S. pneumoniae* in blood by PCR (TAC) with inadequate postmortem lung tissue
  - Detection of *S. pneumoniae* by PCR (TAC) in lung tissue

Level 2

EITHER Moderate histological evidence of pneumonia and detection of *S. pneumoniae* in lung tissue,
OR Pneumonia meeting the clinical criteria for diagnosis of Level 2 pneumonia above, as reported by verbal autopsy, with one of the following:
- Isolation of *S. pneumoniae* from blood culture with inadequate postmortem lung tissue
- Detection of *S. pneumoniae* in blood by PCR (TAC) with inadequate postmortem lung tissue
- Detection of *S. pneumoniae* in lung tissue by PCR (TAC)

Level 3

Detection of *S. pneumoniae* in lung tissue or detection of *S. pneumoniae* in the blood in a patient with a primary respiratory illness but in the absence of sufficient clinical information to meet criteria for Level 1 or Level 2 diagnosis.

Pneumonia due to *Haemophilus influenzae*
ICD-10 Code: J14

Level 1

One of the following:
- Strong histological evidence of pneumonia and detection of *H. influenzae* in lung tissue
- Histological evidence of pneumonia and detection of *H. influenzae* in lung tissue with TWO of the clinical criteria for diagnosis of pneumonia as above, documented in medical record or reported by verbal autopsy.
- Pneumonia meeting TWO of the clinical criteria for diagnosis of pneumonia as above, as documented in the medical record, with one of the following:
  - Isolation of *H. influenzae* from blood culture with inadequate postmortem lung tissue
  - Detection of *H. influenzae* in blood by PCR (TAC) with inadequate postmortem lung tissue
  - Detection of *H. influenzae* in lung tissue by PCR (TAC)

Level 2 EITHER Moderate histological evidence of pneumonia and detection of *H. influenzae* in lung tissue,
OR Pneumonia meeting the clinical criteria for diagnosis of Level 2 pneumonia as above, as reported by verbal autopsy, with one of the following:

- Isolation of *H. influenzae* from blood culture with inadequate postmortem lung tissue
- Detection of *H. influenzae* in blood by PCR (TAC) with inadequate postmortem lung tissue
- Detection of *H. influenzae* in lung tissue by PCR (TAC)

Level 3 Detection of *H. influenzae* in lung tissue or detection of *H. influenzae* in the blood in a patient with a primary respiratory illness but in the absence of sufficient clinical information to meet criteria for Level 1 or Level 2 diagnosis.

Pneumonia due to *Klebsiella pneumoniae*
ICD-10 Code: J15.0

Level 1 One of the following:

- Strong histological evidence of pneumonia and detection of *K. pneumoniae* in lung tissue
- Histological evidence of pneumonia and detection of *K. pneumoniae* in lung tissue with TWO of the clinical criteria for diagnosis of pneumonia as above, documented in medical record or reported by verbal autopsy.
- Pneumonia meeting TWO of the clinical criteria for diagnosis of pneumonia as above, as documented in the medical record, with one of the following:
  - Isolation of *K. pneumoniae* from blood culture with inadequate postmortem lung tissue
  - Detection of *K. pneumoniae* in blood by PCR (TAC) with inadequate postmortem lung tissue
  - Detection of *K. pneumoniae* in lung tissue by PCR (TAC)

Level 2 EITHER Moderate histological evidence of pneumonia and detection of *K. pneumoniae* in lung tissue,
OR Pneumonia meeting TWO of the clinical criteria for diagnosis of Level 2 pneumonia as above, as reported by verbal autopsy, with one of the following:

- Isolation of *K. pneumoniae* from blood culture with inadequate postmortem lung tissue
- Detection of *K. pneumoniae* in blood by PCR (TAC) with inadequate postmortem lung tissue
- Detection of *K. pneumoniae* in lung tissue by PCR (TAC

Level 3 Detection of *K. pneumoniae* in lung tissue or detection of *K. pneumoniae* in the blood in a patient with a primary respiratory illness but in the absence of sufficient clinical information to meet criteria for Level 1 or Level 2 diagnosis.

Pneumonia due to *Pseudomonas aeruginosa*
ICD-10 Code: J15.1

Level 1 One of the following:

- Strong histological evidence of pneumonia and detection of *P. aeruginosa* in lung tissue
- Histological evidence of pneumonia and detection of *P. aeruginosa* in lung tissue with TWO of the clinical criteria for diagnosis of pneumonia as above, documented in medical record or reported by verbal autopsy.
- Pneumonia meeting TWO of the clinical criteria for diagnosis of pneumonia as above, as documented in the medical record, with one of the following:
  - Isolation of *P. aeruginosa* from blood culture with inadequate postmortem lung tissue
  - Detection of *P. aeruginosa* in blood by PCR (TAC) with inadequate postmortem lung tissue
  - Detection of *P. aeruginosa* in lung tissue by PCR (TAC)

Level 2 EITHER Moderate histological evidence of pneumonia and detection of *P. aeruginosa* in lung tissue
OR Pneumonia meeting TWO of the clinical criteria for diagnosis of Level 2 pneumonia above, as reported by verbal autopsy, with one of the following:

- Isolation of *P. aeruginosa* from blood culture with inadequate postmortem lung tissue
- Detection of *P. aeruginosa* in blood by PCR (TAC) with inadequate postmortem lung tissue
- Detection of *P. aeruginosa* in lung tissue by PCR (TAC)

Level 3 Detection *P. aeruginosa* in lung tissue or detection of *P. aeruginosa* in the blood in a patient with a primary respiratory illness but in the absence of sufficient clinical information to meet criteria for Level 1 or Level 2 diagnosis.

Pneumonia due to *Staphylococcus aureus*
ICD-10 Code: J15.2

Level 1 One of the following:

- Strong histological evidence of pneumonia and detection of *P. aeruginosa* in lung tissue
- Histological evidence of pneumonia and detection of *S. aureus* in lung tissue with TWO of the clinical criteria for diagnosis of pneumonia as above, documented in medical record or reported by verbal autopsy.
- Pneumonia meeting TWO of the clinical criteria for diagnosis of pneumonia as above, as documented in the medical record, with one of the following:
  - Isolation of *S. aureus* from blood culture with inadequate postmortem lung tissue
  - Detection of *S. aureus* in blood by PCR (TAC) with inadequate postmortem lung tissue
  - Detection of *S. aureus* in lung tissue by PCR (TAC)

Level 2 EITHER Moderate histological evidence of pneumonia and detection of *S. aureus* in lung tissue
OR Pneumonia meeting TWO of the clinical criteria for diagnosis of Level 2 pneumonia above, as reported by verbal autopsy, with one of the following:

- Isolation of *S. aureus* from blood culture with inadequate postmortem lung tissue
- Detection of *S. aureus* in blood by PCR (TAC) with inadequate postmortem lung tissue
- Detection of *S. aureus* in lung tissue by PCR (TAC)

Pneumonia due to *Streptococcus*, Group B
ICD-10 Code: J15.3

Level 1 One of the following:

- Strong histological evidence of pneumonia and Group B *Streptococcus* in lung tissue
- Histological evidence of pneumonia and Group B *Streptococcus* in lung tissue with TWO of the clinical criteria for diagnosis of pneumonia as above, documented in medical record or reported by verbal autopsy.
- Pneumonia meeting TWO of the clinical criteria for diagnosis of pneumonia as above, as documented in the medical record, with one of the following:
    - Isolation of Group B *Streptococcus* from blood culture with inadequate postmortem lung tissue
    - Detection of Group B *Streptococcus* in blood by PCR (TAC) with inadequate postmortem lung tissue
    - Detection of Group B *Streptococcus* in lung tissue by PCR (TAC)

Level 2 EITHER Moderate histological evidence of pneumonia and detection of Group B *Streptococcus* in lung tissue
OR Pneumonia meeting TWO of the clinical criteria for diagnosis of Level 2 pneumonia above, as reported by verbal autopsy, with one of the following:

- Isolation of Group B *Streptococcus* from blood culture with inadequate postmortem lung tissue
- Detection of Group B *Streptococcus* in blood by PCR (TAC) with inadequate postmortem lung tissue
- Detection of Group B *Streptococcus* in lung tissue by PCR (TAC)

Pneumonia Due to other streptococci (Not Group B or Pneumococcal)
ICD-10 Code: J15.4

Level 1 One of the following:

- Strong histological evidence of pneumonia and detection of Group A or D *Streptococcus* in lung tissue
- Histological evidence of pneumonia and detection of Group A or D *Streptococcus* in lung tissue with TWO of the clinical criteria for diagnosis of pneumonia as above, documented in medical record or reported by verbal autopsy.
- Pneumonia meeting TWO of the clinical criteria for diagnosis of pneumonia as above, as documented in the medical record, with one of the following:
    - Isolation of Group A or D *Streptococcus* from blood culture with inadequate postmortem lung tissue
    - Detection of Group A *Streptococcus* in blood by PCR (TAC) with inadequate postmortem lung tissue
    - Detection of Group A *Streptococcus* in lung tissue by PCR (TAC)

Level 2 EITHER Moderate histological evidence of pneumonia and detection of Group A or D *Streptococcus* in lung tissue
OR Pneumonia meeting TWO of the clinical criteria for diagnosis of Level 2 pneumonia as above, as reported by verbal autopsy, with one of the following:

- Isolation of Group A or D *Streptococcus* from blood culture with inadequate postmortem lung tissue
- Detection of Group A *Streptococcus* in blood by PCR (TAC) with inadequate postmortem lung tissue
- Detection of Group A *Streptococcus* in lung tissue by PCR (TAC)

Pneumonia due to *Escherichia coli*
ICD-10 Code: J15.5

Level 1 One of the following:

- Strong histological evidence of pneumonia and detection of *E. coli* in lung tissue
- Histological evidence of pneumonia and detection of *E. coli* in lung tissue with TWO of the clinical criteria for diagnosis of pneumonia as above, documented in medical record or reported by verbal autopsy.
- Pneumonia meeting TWO of the clinical criteria for diagnosis of pneumonia as above, as documented in the medical record, with one of the following:
    - Isolation of *E. coli* from blood culture with no other pathogen detected and with inadequate postmortem lung tissue
    - Detection of *E. coli* in blood by PCR (TAC) with no other pathogen detected and with inadequate postmortem lung tissue
    - Detection of *E. coli* in lung tissue by PCR (TAC)

Level 2 EITHER Moderate histological evidence of pneumonia and detection of *E. coli* in lung tissue
OR Pneumonia meeting TWO of the clinical criteria for diagnosis of pneumonia as above, as reported by verbal autopsy, with one of the following:

- Isolation of *E. coli* from blood culture with no other pathogen detected and with inadequate postmortem lung tissue

- Detection of *E. coli* in blood by PCR (TAC) with no other pathogen detected and with inadequate postmortem lung tissue
- Detection of *E. coli* in lung tissue by PCR (TAC)

Pneumonia due to other aerobic gram negative bacteria
ICD-10 Code: J15.6

Diagnosis of Level 1 or Level 2 Pneumonia (per the general pneumonia definition above) with one of the following:

- Immunohistochemical (IHC) evidence of a specific aerobic gram negative organism not listed above in lung tissue
- Detection of a specific aerobic gram negative organism not listed above in lung tissue by PCR (TAC) with histological evidence of pneumonia in postmortem biopsy

---

Pneumonia due to *Mycoplasma pneumoniae*
ICD-10 Code: J15.7

Level 1 One of the following:

- Strong histological evidence of pneumonia and detection of *M. pneumoniae* in lung tissue
- Histological evidence of pneumonia and detection of *M. pneumoniae* in lung tissue with TWO of the clinical criteria for diagnosis of pneumonia as above, documented in medical record or reported by verbal autopsy.
- Pneumonia meeting TWO of the clinical criteria for diagnosis of pneumonia as above, as documented in the medical record, with one of the following:
  - Detection of *M. pneumoniae* in NP/OP swab by PCR (TAC)
  - Detection of *M. pneumoniae* in lung tissue by PCR (TAC)

Level 2 EITHER Moderate histological evidence of pneumonia and detection of *M. pneumoniae* in lung tissue
OR Pneumonia meeting TWO of the clinical criteria for diagnosis of pneumonia as above, as reported by verbal autopsy, with one of the following:

- Detection of *M. pneumoniae* in NP/OP swab by PCR (TAC)
- Detection of *M. pneumoniae* in lung tissue by PCR (TAC)

Level 3 Detection of *M. pneumoniae* in the lung tissue or NP/OP swab in the absence of sufficient clinical criteria to meet criteria for Level 1 or Level 2 diagnosis.

Pneumonia due to other specified bacteria
ICD-10 Code: J15.8; J16.8 (if due to bacteria and virus/or other agent together) *Use this code when there is evidence of a specific bacterial etiology but the bacteria does not have an ICD-10 code above. If no bacteria is identified, use J18.*

Diagnosis of Level 1 or Level 2 Pneumonia as noted above with one of the following:

- Immunohistochemical (IHC) evidence of a specific bacteria not listed above in lung tissue
- Detection of a specific bacteria not listed above by TAC (PCR) with histological evidence of pneumonia in postmortem biopsy

---

Influenza with pneumonia
ICD-10 Code: J10.0

Level 1 One of the following:

- Strong histological evidence (diffuse alveolar damage) of pneumonia and detection of influenza virus in lung tissue
- Histological evidence of pneumonia and detection of influenza virus in lung tissue with acute respiratory illness with fever and cough, documented in medical record or reported by verbal autopsy.
- Acute respiratory illness with fever and cough, as documented in the medical record, with detection of influenza virus in lung tissue by PCR (TAC)

Level 2 EITHER Moderate histological evidence of pneumonia and detection of influenza virus in lung tissue OR
acute respiratory illness with fever and cough, as reported by verbal autopsy, with detection of influenza virus in lung tissue by PCR (TAC).

Influenza with other respiratory manifestations (not pneumonia)
ICD-10 Code: J10.1

If pneumonia is present, use the above diagnosis standard.

Level 1 Detection of influenza virus in NP/OP swab or Lung Tissue by PCR (TAC) AND acute respiratory illness with fever and cough documented in medical record, without pathologic evidence of pneumonia.

Level 2 Detection of influenza virus in NP/OP swab or Lung Tissue by PCR (TAC) AND acute respiratory illness with fever and cough as reported by verbal

autopsy, without pathologic evidence of pneumonia.

Level 3 Acute respiratory illness with fever or hypothermia with tachypnea, respiratory distress, cough, abnormal breath sounds, hypoxia or cyanosis documented in the medical record or reported by verbal autopsy, and detection of influenza virus in NP/OP swab by PCR (TAC).

Pneumonia due to Adenovirus
ICD-10 Code: J12.0

Level 1 One of the following:

- Strong histological evidence of viral pneumonia and detection of adenovirus virus in lung tissue by PCR (TAC)
- Histological evidence of pneumonia and detection of adenovirus virus in lung tissue by PCR (TAC) with TWO of the clinical criteria for diagnosis of pneumonia as above, documented in medical record or reported by verbal autopsy.
- Pneumonia meeting TWO of the clinical criteria for diagnosis of pneumonia as above, as documented in the medical record, with detection of adenovirus in lung tissue by PCR (TAC)

Level 2 Pneumonia meeting TWO of the clinical criteria for diagnosis of Level 2 pneumonia above, as reported by verbal autopsy, with detection of adenovirus in lung tissue by PCR (TAC).

Level 3 One of the following:

- Acute febrile illness or hypothermia with tachypnea, respiratory distress, abnormal breath sounds, hypoxia or cyanosis documented in the medical record or reported by verbal autopsy, and detection of adenovirus in NP/OP swab by PCR (TAC).
- Detection of adenovirus in the lung tissue in the absence of sufficient clinical information for Level 1 or Level 2 diagnosis.

Pneumonia due to Respiratory Syncytial Virus (RSV)
ICD-10 Code: J12.1

Level 1 One of the following:

- Strong histological evidence of viral pneumonia and detection of RSV in lung tissue by PCR (TAC) or immunohistochemistry (IHC).
- Histological evidence of pneumonia and detection of RSV in lung tissue by PCR (TAC) or immunohistochemistry (IHC) with TWO of the clinical criteria for diagnosis of pneumonia as above, documented in medical record or reported by verbal autopsy.
- Pneumonia meeting TWO of the clinical criteria for diagnosis of pneumonia as above, as documented in the medical record, with detection of RSV in lung tissue by PCR (TAC)

Level 2 Pneumonia meeting TWO of the clinical criteria for diagnosis of Level 2 pneumonia above, as reported by verbal autopsy, with detection of RSV in lung tissue by PCR (TAC).

Level 3 One of the following:

- Acute febrile illness or hypothermia with tachypnea, respiratory distress, abnormal breath sounds, hypoxia or cyanosis documented in the medical record or reported by verbal autopsy, and detection of RSV in NP/OP swab by PCR (TAC).
- Detection of RSV in the lung tissue by PCR (TAC) in the absence of sufficient information for Level 1 or Level 2 diagnosis.

Pneumonia due to Parainfluenza
ICD-10 Code: J12.2

Level 1 One of the following:

- Strong histological evidence of viral pneumonia and detection of parainfluenza virus in lung tissue by PCR (TAC)
- Histological evidence of pneumonia and detection of parainfluenza virus in lung tissue by PCR (TAC) with TWO of the clinical criteria for diagnosis of pneumonia as above, documented in medical record or reported by verbal autopsy.
- Pneumonia meeting TWO of the clinical criteria for diagnosis of pneumonia as above, as documented in the medical record, with detection of parainfluenza virus in lung tissue by PCR (TAC).

Level 2 Pneumonia meeting TWO of the clinical criteria for diagnosis of Level 2 pneumonia above, as reported by verbal autopsy, with detection of parainfluenza virus in lung tissue by PCR (TAC).

Level 3 One of the following:

- Acute febrile illness or hypothermia with tachypnea, respiratory distress, abnormal breath sounds, hypoxia or cyanosis documented in the medical record or reported by verbal autopsy, and detection of parainfluenza virus in NP/OP swab by PCR (TAC).
- Detection of parainfluenza virus in the lung tissue by PCR (TAC) in the absence of sufficient clinical information for Level 1 or Level 2 diagnosis.

Pneumonia due to Human Metapneumovirus (HMPV)
ICD-10: J12.3

Level 1 One of the following:

- Strong histological evidence of viral pneumonia and detection of HMPV in lung tissue by PCR (TAC) or immunohistochemistry (IHC).

- Histological evidence of pneumonia and detection of HMPV in lung tissue by PCR (TAC) with TWO of the clinical criteria for diagnosis of pneumonia as above, documented in medical record or reported by verbal autopsy.
- Pneumonia meeting TWO of the clinical criteria for diagnosis of pneumonia as above, as documented in the medical record, with detection of HMPV in lung tissue by PCR (TAC).

Level 2 Pneumonia meeting TWO of the clinical criteria for diagnosis of Level 2 pneumonia above, as reported by verbal autopsy, with detection of HMPV in lung tissue by PCR (TAC).

Level 3 One of the following:

- Acute febrile illness or hypothermia with tachypnea, respiratory distress, abnormal breath sounds, hypoxia or cyanosis documented in the medical record or reported by verbal autopsy, and detection of HMPV in NP/OP swab by PCR (TAC).
- Detection of HMPV in the lung tissue by PCR (TAC) in the absence of sufficient clinical information for Level 1 or Level 2 diagnosis.

Pneumonia due to Other Specified Virus
ICD-10 Code: J12.9
Use this code when a specific etiology is identified but does not have an ICD-10 code above.

Level 1 One of the following:

- Strong histological evidence of viral pneumonia and detection of the virus in lung tissue by PCR (TAC) or immunohistochemistry (IHC).
- Histological evidence of pneumonia and detection of the virus in lung tissue by PCR (TAC) with TWO of the clinical criteria for diagnosis of pneumonia as above, documented in medical record or reported by verbal autopsy.
- Pneumonia meeting TWO of the clinical criteria for diagnosis of pneumonia as above, as documented in the medical record, with detection of the virus in lung tissue by PCR (TAC).

Level 2 Pneumonia meeting TWO of the clinical criteria for diagnosis of Level 2 pneumonia above, as reported by verbal autopsy, with detection of the virus in lung tissue by PCR (TAC).

Level 3 One of the following:

- Acute febrile illness or hypothermia with tachypnea, respiratory distress, abnormal breath sounds, hypoxia or cyanosis documented in the medical record or reported by verbal autopsy, and detection of the virus in NP/OP swab by PCR (TAC).
- Detection of the virus in the lung tissue by PCR (TAC) in the absence of sufficient clinical information for Level 1 or Level 2 diagnosis.

Pneumonia due to *Chlamydia*
ICD-10 Code: J16.0

Level 1 One of the following:

- Strong Histological evidence of pneumonia and detection of *C. pneumoniae* (at all ages) or *C. trachomatis* (infants <6 months) in lung tissue by PCR (TAC)
- Histological evidence of pneumonia and detection of *C. pneumoniae* (at all ages) or *C. trachomatis* (infants <6 months) in lung tissue by PCR (TAC) with TWO of the clinical criteria for diagnosis of pneumonia as above, documented in medical record or reported by verbal autopsy.
- Pneumonia meeting TWO of the clinical criteria for diagnosis of pneumonia as above, as documented in the medical record, with detection of *C. pneumoniae* (at all ages) or *C. trachomatis* (infants <6 months) in lung tissue by PCR (TAC).

Level 2 Pneumonia meeting TWO of the clinical criteria for diagnosis of Level 2 pneumonia above, as reported by verbal autopsy, with detection of *C. pneumoniae* (at all ages) or *C. trachomatis* (infants <6 months) in lung tissue by PCR (TAC).

Level 3 One of the following:

- Acute febrile illness or hypothermia with tachypnea, respiratory distress, abnormal breath sounds, hypoxia or cyanosis documented in the medical record or reported by verbal autopsy, with detection of *C. pneumoniae* (at all ages) or *C. trachomatis* (infants <6 months) in NP/OP swab by PCR (TAC).
- Detection of *C. pneumoniae* (at all ages) or *C. trachomatis* (infants <6 months) in lung tissue by PCR (TAC) in the absence of sufficient clinical information for Level 1 or Level 2 diagnosis.

Pneumonia due to *Pneumocystis* (PCP)
ICD-10 Code: B59 AND J17.3

Level 1: Either of the following findings in an immunocompromised, severely malnourished, or HIV+ patient:

- Immunohistochemical (IHC) evidence of PCP in lung tissue
- Detection of PCP in lung tissue by PCR (TAC) with histological evidence of pneumonia in postmortem biopsy

Level 3: Either of the following findings in an immunocompromised, severely malnourished, or HIV+ patient:

- Chest radiograph with “ground glass” infiltrates without known alternative etiology and without sufficient lung tissue for analysis
- Detection of PCP by PCR in lung tissue

Pneumonia due to other infectious organism, not elsewhere classified
ICD-10 Code: J15

Diagnosis of Level 1 or Level 2 Pneumonia as noted above with one of the following:

- Immunohistochemical (IHC) evidence of non-bacterial pathogen not listed above in lung tissue
- Detection of a non-bacterial pathogen in lung tissue by PCR (TAC) with histological evidence of pneumonia in postmortem biopsy

Supplemental Table S3. Causes of death in the causal chain for all 1120 deaths including those without pneumonia, CHAMPS Network, Africa and South Asia, December 16th 2016 to December 31st, 2022.

Cause of Death	N (%)
Lower respiratory infections	455 (40.6)
Sepsis	424 (37.9)
Malnutrition	278 (24.8)
Malaria	248 (22.1)
Anemias	186 (16.6)
Diarrheal Diseases	163 (14.6)
HIV	109 (9.7)
Congenital birth defects	97 (8.7)
Other respiratory disease	91 (8.1)
Meningitis/Encephalitis	82 (7.3)
Other infections	65 (5.8)
Neonatal preterm birth complications	59 (5.3)
Injury	55 (4.9)
Undetermined	44 (3.9)
Other	36 (3.2)
Other neonatal disorders	24 (2.1)
Other neurological disorders	23 (2.1)
Liver disease	16 (1.4)
Other endocrine, metabolic, blood, and immune disorders	16 (1.4)
Poisoning	15 (1.3)
Tuberculosis	14 (1.2)
Sickle cell disorders	13 (1.2)
Heart Diseases	11 (1.0)
Congenital infection	9 (0.8)
Other disorders of fluid, electrolyte and acid-base balance	9 (0.8)
Paralytic ileus and intestinal obstruction	9 (0.8)
Cancer	8 (0.7)
Other skin and subcutaneous diseases	7 (0.6)
Sudden infant death syndrome	6 (0.5)
Epilepsy	5 (0.4)
Kidney Disease	5 (0.4)
Other immunodeficiencies	5 (0.4)
Measles	4 (0.4)
Syphilis	4 (0.4)
Upper respiratory infections	4 (0.4)
Neonatal encephalopathy	2 (0.2)
Perinatal asphyxia/hypoxia	2 (0.2)
Rabies	2 (0.2)
Diabetes	1 (0.1)
Motor neuron disease	1 (0.1)
Other gastrointestinal disease	1 (0.1)

Supplemental Table S4. Ethnicities* of deceased children aged 1-59 months by whether pneumonia was attributed as a cause in the pathway leading to death (N = 1120), CHAMPS Network, Africa and South Asia, December 16th, 2016, to December 31st, 2022.

	Overall (N = 1120)	Deaths from causes other than pneumonia (N = 6655)	Deaths attributed to pneumonia (N = 455)
	**N (%)**	**N (%)**	**N (%)**
**Bangladesh**			
Bangladeshi	6 (100)	4 (100)	2 (100)
**Ethiopia**			
Oromo	25 (96.2)	6 (100)	19 (95.0)
Missing	1 (3.8)	0 (0)	1 (5.0)
**Kenya**			
Luo	154 (57.0)	115 (59.9)	39 (50.0)
Luhya	2 (0.7)	2 (1.0)	0 (0)
Kisii	2 (0.7)	2 (1.0)	0 (0)
Kikuyu	1 (0.4)	0 (0)	1 (1.3)
Other race or ethnicity	14 (5.2)	11 (5.7)	3 (3.8)
Missing	97 (35.9)	62 (32.3)	35 (44.9)
**Mali**			
Bambara	15 (25.0)	7 (23.3)	8 (26.7)
Malinke	19 (31.7)	10 (33.3)	9 (30.0)
Sonike	6 (10.0)	2 (6.7)	4 (13.3)
Peuhl	5 (8.3)	3 (10.0)	2 (6.7)
Bobo	1 (1.7)	1 (3.3)	0 (0)
Senoufo	3 (5.0)	1 (3.3)	2 (6.7)
Bozo	1 (1.7)	0 (0)	1 (3.3)
Somono	1 (1.7)	0 (0)	1 (3.3)
Dogon	1 (1.7)	1 (3.3)	0 (0)
Sonrhai	2 (3.3)	1 (3.3)	1 (3.3)
Tamachek	1 (1.7)	1 (3.3)	0 (0)
Other race or ethnicity	3 (5.0)	2 (6.7)	1 (3.3)
Missing	2 (3.3)	1 (3.3)	1 (3.3)
**Mozambique**			
Other race or ethnicity	69 (36.1)	38 (37.6)	31 (34.4)
Missing	122 (63.9)	63 (62.4)	59 (65.6)
**Sierra Leone**			
Themne	164 (58.0)	118 (59.3)	46 (54.8)
Mende	73 (25.8)	48 (24.1)	25 (29.8)
Limba	5 (1.8)	3 (1.5)	2 (2.4)
Creole	2 (0.7)	2 (1.0)	0 (0)
Mandingo	6 (2.1)	2 (1.0)	4 (4.8)
Fullah	12 (4.2)	9 (4.5)	3 (3.6)
Susu	1 (0.4)	1 (0.5)	0 (0)
Kurankoh	3 (1.1)	3 (1.5)	0 (0)
Other race or ethnicity	2 (0.7)	2 (1.0)	0 (0)
Missing	15 (5.3)	11 (1.7)	4 (4.8)
**South Africa**			
Black Africa	279 (98.2)	132 (99.2)	147 (97.4)
Other race or ethnicity	5 (1.8)	1 (0.8)	4 (2.6)

* Reported from family or caregiver.

**Supplemental Table S5:** Characteristics of deaths attributed to pneumonia, by CHAMPS site							
	Bangladesh	Ethiopia	Kenya	Mali	Mozambique	Sierra Leone	South Africa
Characteristics	N=2	N=20	N=78	N=30	N= 90	N=84	N=151
Age at death (N, %)							
Early infants (1 month to >6months)	0 (0.0)	7 (35.0)	23 (29.5)	10 (33.3)	20 (22.2)	22 (26.2)	88 (58.3)
Late infants (≥6 months to > 12 months)	0 (0.0)	2 (10.0)	24 (30.8)	6 (20.0)	17 (18.9)	20 (23.8)	25 (16.6)
Childrens (≥12 months to >60months)	2 (100.0)	11 (55.0)	31 (39.7)	14 (46.7)	53 (58.9)	42 (50.0)	38 (25.2)
Median age (range), in months	22 [20, 25]	17 [3, 21]	9 [6, 20]	11 [6, 18]	15 [7, 25]	12 [6, 17]	4 [2, 12]
Male sex (N, %)	2 (100.0)	12 (60.0)	45 (57.7)	15 (50.0)	59 (65.6)	45 (53.6)	87 (57.6)
HIV status, n (%)							
HIV infected	0 (0.0)	0 (0.0)	11 (14.1)	3 (10.0)	22 (24.4)	8 (9.5)	27 (17.9)
HIV exposed uninfected	0 (0.0)	0 (0.0)	11 (14.1)	0 (0.0)	12 (13.3)	1 (1.2)	36 (23.8)
HIV uninfected*	2 (100.0)	20 (100.0)	56 (71.8)	27 (90.0)	56 (62.2)	75 (89.3)	88 (58.3)
Weight for height z-score (N,%)							
Normal weight for height (≥ -2SD)	0 (0.0)	8 (40.0)	23 (29.5)	4 (13.3)	46 (51.1)	25 (29.8)	69 (45.7)
Moderate wasting (<-2 SD, -3 SD)	0 (0.0)	5 (25.0)	11 (14.1)	3 (10.0)	10 (11.1)	13 (15.5)	14 (9.3)
Severe wasting (<-3 SD)	2 (100.0)	5 (25.0)	42 (53.8)	23 (76.7)	30 (33.3)	43 (51.2)	44 (29.1)
Not recorded	0 (0.0)	2 (10.0)	2 (2.6)	0 (0.0)	4 (4.4)	3 (3.6)	24 (15.9)
Weight for age z-score (N, %)							
Normal weight for age (≥ -2SD)	0 (0.0)	4 (20.0)	22 (28.2)	8 (26.7)	39 (43.3)	24 (28.6)	57 (37.7)
Moderate underweight (<-2 SD , -3 SD)	1 (50.0)	0 (0.0)	13 (16.7)	7 (23.3)	13 (14.4)	17 (20.2)	17 (11.3)
Severe underweight (< -3 SD)	1 (50.0)	16 (80.0)	43 (55.1)	14 (46.7)	38 (42.2)	43 (51.2)	71 (47.0)
Not recorded	0 (0.0)	0 (0.0)	0 (0.0)	1 (3.3)	0 (0.0)	0 (0.0)	6 (4.0)
Height for age z score (N, %)							
Normal height for age (≥ -2SD)	1 (50.0)	5 (25.0)	44 (56.4)	23 (76.7)	42 (46.7)	55 (65.5)	75 (49.7)
Moderate stunting (<-2 SD, -3 SD)	1 (50.0)	1 (5.0)	12 (15.4)	1 (3.3)	10 (11.1)	12 (14.3)	25 (16.6)
Severe stunting (<- 3 SD)	0 (0.0)	14 (70.0)	22 (28.2)	5 (16.7)	38 (42.2)	17 (20.2)	49 (32.5)
Not recorded	0 (0.0)	0 (0.0)	0 (0.0)	1 (3.3)	0 (0.0)	0 (0.0)	2 (1.3)
Median time between death and MITS done (hours)	3 [2, 3]	7 [2, 14]	15 [10, 23]	10 [4, 13]	11 [4, 19]	7 [3, 12]	22 [17, 36]
Location of death (N, %)							
Community	0 (0)	10 (50.0)	36 (46.2)	10 (33.3)	20 (22.2)	6 (7.1)	17 (11.3)
Health facility	2 (100.0)	10 (50.0)	42 (53.8)	20 (66.7)	70 (77.8)	78 (92.9)	134 (88.7)
For health facility deaths**							
Median number days between admission & death	8 [5, 10]	2 [1, 4]	1 [0, 3]	1 [0, 1]	1 [0, 5]	1 [0, 3]	6 [2, 31]

*Data were missing on confirmed lack of exposure to HIV, therefore we reported these deaths as HIV uninfected

**Number of deaths happened in health facilities were used as a denominator for median number of days between admission and death.

Supplemental Table S6: Other conditions in the causal pathway to death when pneumonia is one of the conditions in the causal pathway stratified by age group and by whether the pneumonia death 1) occurred in the community or with fewer than 72 hours in the hospital or 2) occurred 72 or more hours after hospital admission, CHAMPS Network, December 16th 2016 to December 31st, 2022.

	**Community deaths or <72 hours of hospital stay**			**Hospital-associated (i.e. death at ≥72 hours after admission to hospital)**		
	1-6 months	6-12 months	12-59 months	1-6 months	6-12 months	12-59 months
**Cause of death***	N = 104	N = 66	N = 136	N = 66	N = 28	N = 55
Sepsis	47 (45.2)	30 (45.5)	51 (37.5)	34 (51.5)	12 (42.9)	26 (47.3)
Malnutrition	19 (18.3)	32 (48.5)	52 (38.2)	6 (9.1)	7 (25.0)	12 (21.8)
HIV	13 (12.5)	5 (7.6)	19 (14.0)	11 (16.7)	4 (14.3)	14 (25.5)
Anemias	4 (3.8)	10 (15.2)	28 (20.6)	5 (7.6)	5 (17.9)	9 (16.4)
Diarrheal Diseases	7 (6.7)	12 (18.2)	22 (16.2)	6 (9.1)	5 (17.9)	6 (10.9)
Congenital birth defects	12 (11.5)	5 (7.6)	11 (8.1)	16 (24.2)	2 (7.1)	9 (16.4)
Meningitis/Encephalitis	10 (9.6)	7 (10.6)	10 (7.4)	14 (21.2)	2 (7.1)	9 (16.4)
Malaria	4 (3.8)	5 (7.6)	21 (15.4)	0 (0)	5 (17.9)	3 (5.5)
Neonatal preterm birth complications	9 (8.7)	3 (4.5)	1 (0.7)	14 (21.2)	2 (7.1)	0 (0)
Other infections	5 (4.8)	2 (3.0)	11 (8.1)	9 (13.6)	0 (0)	2 (3.6)
Other respiratory disease	1 (1.0)	0 (0)	4 (2.9)	4 (6.1)	4 (14.3)	6 (10.9)
Injury	1 (1.0)	0 (0)	4 (2.9)	0 (0)	1 (3.6)	11 (20.0)
Other	3 (2.9)	3 (4.5)	2 (1.5)	4 (6.1)	0 (0)	2 (3.6)
Other neurological disorders	2 (1.9)	1 (1.5)	4 (2.9)	2 (3.0)	2 (7.1)	2 (3.6)
Other neonatal disorders	2 (1.9)	0 (0)	0 (0)	8 (12.1)	1 (3.6)	0 (0)
Other endocrine, metabolic, blood, and immune disorders	2 (1.9)	2 (3.0)	2 (1.5)	2 (3.0)	1 (3.6)	0 (0)
Heart Diseases	1 (1.0)	1 (1.5)	0 (0)	3 (4.5)	0 (0)	0 (0)
Congenital infection	1 (1.0)	0 (0)	1 (0.7)	2 (3.0)	0 (0)	0 (0)
Kidney Disease	0 (0)	0 (0)	1 (0.7)	2 (3.0)	1 (3.6)	0 (0)
Liver disease	1 (1.0)	0 (0)	2 (1.5)	0 (0)	0 (0)	1 (1.8)
Other skin and subcutaneous diseases	1 (1.0)	1 (1.5)	1 (0.7)	0 (0)	0 (0)	1 (1.8)
Sickle cell disorders	0 (0)	0 (0)	3 (2.2)	0 (0)	0 (0)	1 (1.8)
Tuberculosis	0 (0)	0 (0)	0 (0)	0 (0)	0 (0)	4 (7.3)
Measles	0 (0)	0 (0)	3 (2.2)	0 (0)	0 (0)	0 (0)
Other disorders of fluid, electrolyte and acid-base balance	0 (0)	0 (0)	0 (0)	1 (1.5)	2 (7.1)	0 (0)
Upper respiratory infections	1 (1.0)	1 (1.5)	1 (0.7)	0 (0)	0 (0)	0 (0)
Other immunodeficiencies	0 (0)	0 (0)	1 (0.7)	1 (1.5)	0 (0)	0 (0)
Cancer	0 (0)	0 (0)	0 (0)	0 (0)	0 (0)	1 (1.8)
Motor neuron disease	0 (0)	0 (0)	0 (0)	1 (1.5)	0 (0)	0 (0)
Neonatal encephalopathy	1 (1.0)	0 (0)	0 (0)	0 (0)	0 (0)	0 (0)
Perinatal asphyxia/hypoxia	0 (0)	0 (0)	0 (0)	1 (1.5)	0 (0)	0 (0)
Poisoning	0 (0)	0 (0)	0 (0)	0 (0)	1 (3.6)	0 (0)
Syphilis	0 (0)	0 (0)	0 (0)	1 (1.5)	0 (0)	0 (0)

*Conditions in the causal pathway were assigned as per CHAMPS-developed diagnosis standards available at https://champshealth.org/wp-content/uploads/2021/01/CHAMPS-Diagnosis-Standards.pdf

**Supplemental Table S7**. Pathogens in the causal chain for all 1120 deaths including those without pneumonia, CHAMPS Network, Africa and South Asia, December 16th 2016 to December 31st, 2022.	
**Pathogen**	**N (%)**
Klebsiella pneumoniae	283 (25.3)
Plasmodium falciparum	238 (21.2)
Streptococcus pneumoniae	154 (13.8)
Human Immunodeficiency Virus	108 (9.6)
Escherichia coli	105 (9.4)
Cytomegalovirus	92 (8.2)
Acinetobacter baumannii	67 (6.0)
Non-typeable *Haemophilus influenzae*	63 (5.6)
Staphylococcus aureus	55 (4.9)
Adenovirus	46 (4.1)
Pseudomonas aeruginosa	43 (3.8)
Streptococcus spp.	40 (3.6)
Pneumocystis jirovecii	38 (3.4)
Respiratory syncytial virus	31 (2.8)
Enterococcus faecium	24 (2.1)
Enterococcus faecalis	21 (1.9)
Candida albicans	18 (1.6)
Haemophilus influenzae Type A	17 (1.5)
Salmonella spp.	17 (1.5)
Moraxella catarrhalis	16 (1.4)
Rotavirus A	14 (1.2)
Mycobacterium tuberculosis	13 (1.2)
Candida spp.	12 (1.1)
Parainfluenza virus type 3	11 (1.0)
Rhinovirus	11 (1.0)
Influenza A	10 (0.9)
Rotavirus non-typable	10 (0.9)
Streptococcus pyogenes	10 (0.9)
Escherichia coli/Shigella spp.	9 (0.8)
Human metapneumovirus	8 (0.7)
Bordetella pertussis	7 (0.6)
Streptococcus viridans	7 (0.6)
Campylobacter jejuni	6 (0.5)
Candida parapsilosis	6 (0.5)
Enterobacter cloacae	6 (0.5)
Influenza B	6 (0.5)
Klebsiella spp.	5 (0.4)
Parainfluenza virus type 1	5 (0.4)
Streptococcus agalactiae	5 (0.4)
Other*	82 (7.3)

* Other includes Adenovirus 40/41, Enterovirus, *Haemophilus influenzae* Type B, Measles, *Neisseria meningitidis*, *Shigella spp*., *Treponema pallidum*, Norovirus GI, Parainfluenza virus type 4, *Vibrio cholerae*, Astrovirus, *Bordetella spp*., *Candida auris, Cryptosporidium parvum, Enterococcus spp., Haemophilus parainfluenzae, Haemophilus spp*., Herpes simplex virus 1, Parvovirus B19, SARS-CoV-2, Sapovirus, *Aspergillus*, *Brevundimonas vesicularis*, *Brucella spp*., *Candida krusei*, *Candida tropicalis, Chlamydia trachomatis, Giardia spp., Haemophilus aphrophilus,* Hepatitis C, Human coronavirus OC43, *Klebsiella ornithinolytica, Klebsiella oxytoca*, Lassa Fever Virus, *Morganella morganii*, Norovirus GII, *Ochrobactrum anthropi*, Parainfluenza virus type 2, *Pneumocystis spp., Proteus mirabilis, Proteus vulgaris, Pseudomonas stuartii, Serratia marcescens, Serratia odorifera, Serratia spp., Staphylococcus epidermidis, Staphylococcus haemolyticus, Stenotrophomonas maltophilia, Toxoplasma gondii, Ureaplasma spp*

**Supplemental Table S8.** Pathogens identified as causing pneumonia deaths by age group, CHAMPS Network, December 16th 2016 to December 31st, 2022.				
	All	1-6 months	6-12 months	12-59 months
	N = 455	N = 170	N = 94	N = 191
**Pathogen**	n (%)	n (%)	n (%)	n (%)
**Gram negative bacteria**	**232 (51.0)**	**96 (56.5)**	**40 (42.6)**	**96 (50.3)**
Klebsiella pneumoniae	142 (31.2)	63 (37.1)	23 (24.5)	56 (29.3)
Non-typeable *Haemophilus influenzae*	45 (9.9)	11 (6.5)	9 (9.6)	25 (13.1)
Acinetobacter baumannii	30 (6.6)	20 (11.8)	4 (4.3)	6 (3.1)
Pseudomonas aeruginosa	25 (5.5)	8 (4.7)	4 (4.3)	13 (6.8)
Escherichia coli	16 (3.5)	7 (4.1)	3 (3.2)	6 (3.1)
Haemophilus influenzae Type A	16 (3.5)	3 (1.8)	3 (3.2)	10 (5.2)
Moraxella catarrhalis	13 (2.9)	4 (2.4)	3 (3.2)	6 (3.1)
Bordetella pertussis	7 (1.5)	7 (4.1)	0 (0)	0 (0)
Haemophilus influenzae Type B	3 (0.7)	2 (1.2)	1 (1.1)	0 (0)
Klebsiella spp.	3 (0.7)	0 (0)	2 (2.1)	1 (0.5)
Salmonella spp.	3 (0.7)	0 (0)	1 (1.1)	2 (1.0)
Bordetella spp.	2 (0.4)	1 (0.6)	0 (0)	1 (0.5)
Haemophilus parainfluenzae	2 (0.4)	1 (0.6)	0 (0)	1 (0.5)
Haemophilus spp.	2 (0.4)	0 (0)	1 (1.1)	1 (0.5)
Chlamydia trachomatis	1 (0.2)	1 (0.6)	0 (0)	0 (0)
Haemophilus aphrophilus	1 (0.2)	1 (0.6)	0 (0)	0 (0)
Ureaplasma spp.	1 (0.2)	1 (0.6)	0 (0)	0 (0)
**Gram positive bacteria**	**182 (40.0)**	**61 (35.9)**	**41 (43.6)**	**80 (41.9)**
Streptococcus pneumoniae	123 (27.0)	33 (19.4)	33 (35.1)	57 (29.8)
Staphylococcus aureus	31 (6.8)	16 (9.4)	4 (4.3)	11 (5.8)
Streptococcus spp.	26 (5.7)	9 (5.3)	5 (5.3)	12 (6.3)
Streptococcus viridans	4 (0.9)	2 (1.2)	1 (1.1)	1 (0.5)
Enterococcus faecalis	3 (0.7)	1 (0.6)	0 (0)	2 (1.0)
Streptococcus agalactiae	3 (0.7)	1 (0.6)	0 (0)	2 (1.0)
Streptococcus pyogenes	2 (0.4)	2 (1.2)	0 (0)	0 (0)
Enterococcus faecium	1 (0.2)	1 (0.6)	0 (0)	0 (0)
Enterococcus spp.	1 (0.2)	0 (0)	0 (0)	1 (0.5)
**Virus**	**145 (31.9)**	**67 (39.4)**	**32 (34.0)**	**46 (24.1)**
Cytomegalovirus	54 (11.9)	29 (17.1)	13 (13.8)	12 (6.3)
Respiratory syncytial virus	29 (6.4)	17 (10.0)	4 (4.3)	8 (4.2)
Adenovirus	25 (5.5)	10 (5.9)	6 (6.4)	9 (4.7)
Influenza A	10 (2.2)	1 (0.6)	3 (3.2)	6 (3.1)
Parainfluenza virus type 3	10 (2.2)	5 (2.9)	2 (2.1)	3 (1.6)
Rhinovirus	10 (2.2)	6 (3.5)	1 (1.1)	3 (1.6)
Human metapneumovirus	7 (1.5)	3 (1.8)	1 (1.1)	3 (1.6)
Influenza B	6 (1.3)	2 (1.2)	2 (2.1)	2 (1.0)
Parainfluenza virus type 1	5 (1.1)	0 (0)	3 (3.2)	2 (1.0)
Parainfluenza virus type 4	3 (0.7)	0 (0)	2 (2.1)	1 (0.5)
Human coronavirus OC43	1 (0.2)	1 (0.6)	0 (0)	0 (0)
Parainfluenza virus type 2	1 (0.2)	1 (0.6)	0 (0)	0 (0)
SARS-CoV-2	1 (0.2)	0 (0)	0 (0)	1 (0.5)
**Fungus**	**35 (7.7)**	**22 (12.9)**	**5 (5.3)**	**8 (4.2)**
Pneumocystis jirovecii	27 (5.9)	19 (11.2)	3 (3.2)	5 (2.6)
Candida albicans	3 (0.7)	0 (0)	0 (0)	3 (1.6)
Candida spp.	3 (0.7)	2 (1.2)	1 (1.1)	0 (0)
Candida auris	1 (0.2)	0 (0)	1 (1.1)	0 (0)
Pneumocystis spp.	1 (0.2)	1 (0.6)	0 (0)	0 (0)
**No pathogen implicated**	78 (17.1)	22 (12.9)	17 (18.1)	39 (20.4)

Supplemental Table S9. Pathogens identified as causing pneumonia deaths, for all pneumonia deaths and stratified by whether the pneumonia was the underlying, immediate, or antecedent cause of death and by whether the pneumonia death 1) occurred in the community or with fewer than 72 hours in the hospital or 2) 72 or more hours after hospital admission, CHAMPS Network, December 16th 2016 to December 31st, 2022.

	**Community deaths or <72 hours of hospital stay**				**Hospital-associated (i.e. deaths occurring ≥72 hours after admission to hospital)**			
	All pneumonia deaths	Pneumonia was underlying cause of death	Pneumonia was immediate cause of death	Pneumonia was antecedent cause of death	All pneumonia deaths	Pneumonia was underlying cause of death	Pneumonia was immediate cause of death	Pneumonia was antecedent cause of death
	N = 306[b]	N = 88	N = 128	N = 99	N = 149[a]	N = 17	N = 73	N = 65
**Pathogen**	n (%)	n (%)	n (%)	n (%)	n (%)	n (%)	n (%)	n (%)
**Gram negative bacteria**	**148 (48.4)**	**37 (42.0)**	**60 (46.9)**	**53 (53.5)**	**84 (56.4)**	**8 (47.1)**	**35 (47.9)**	**42 (64.6)**
Klebsiella pneumoniae	78 (25.5)	15 (17.0)	29 (22.7)	34 (34.3)	64 (43.0)	5 (29.4)	23 (31.5)	36 (55.4)
Non-typeable *Haemophilus influenzae*	37 (12.1)	7 (8.0)	19 (14.8)	11 (11.1)	8 (5.4)	2 (11.8)	4 (5.5)	2 (3.1)
Acinetobacter baumannii	11 (3.6)	1 (1.1)	4 (3.1)	6 (6.1)	19 (12.8)	1 (5.9)	7 (9.6)	11 (16.9)
Pseudomonas aeruginosa	10 (3.3)	1 (1.1)	2 (1.6)	7 (7.1)	15 (10.1)	0 (0)	7 (9.6)	8 (12.3)
Escherichia coli	14 (4.6)	1 (1.1)	5 (3.9)	8 (8.1)	2 (1.3)	0 (0)	1 (1.4)	1 (1.5)
Haemophilus influenzae type A	14 (4.6)	7 (8.0)	6 (4.7)	1 (1.0)	2 (1.3)	0 (0)	2 (2.7)	0 (0)
Moraxella catarrhalis	12 (3.9)	3 (3.4)	7 (5.5)	2 (2.0)	1 (0.7)	0 (0)	1 (1.4)	0 (0)
Bordetella pertussis	4 (1.3)	4 (4.5)	0 (0)	0 (0)	3 (2.0)	1 (5.9)	3 (4.1)	0 (0)
Haemophilus influenzae Type B	3 (1.0)	2 (2.3)	0 (0)	1 (1.0)	0 (0)	0 (0)	0 (0)	0 (0)
Klebsiella spp.[b]	2 (0.7)	1 (1.1)	1 (0.8)	0 (0)	1 (0.7)	0 (0)	1 (1.4)	0 (0)
Salmonella spp.	3 (1.0)	0 (0)	2 (1.6)	1 (1.0)	0 (0)	0 (0)	0 (0)	0 (0)
Haemophilus parainfluenzae	2 (0.7)	0 (0)	1 (0.8)	1 (1.0)	0 (0)	0 (0)	0 (0)	0 (0)
Chlamydia trachomatis	1 (0.3)	0 (0)	1 (0.8)	0 (0)	0 (0)	0 (0)	0 (0)	0 (0)
Haemophilus aphrophilus	0 (0)	0 (0)	0 (0)	0 (0)	1 (0.7)	0 (0)	1 (1.4)	0 (0)
Haemophilus spp.	1 (0.3)	1 (1.1)	0 (0)	1 (1.0)	0 (0)	0 (0)	0 (0)	0 (0)
Ureaplasma spp.	0 (0)	0 (0)	0 (0)	0 (0)	1 (0.7)	0 (0)	1 (1.4)	0 (0)
**Gram positive bacteria**	**142 (46.4)**	**35 (39.8)**	**58 (45.3)**	**50 (50.5)**	**40 (26.8)**	**1 (5.9)**	**23 (31.5)**	**16 (24.6)**
Streptococcus pneumoniae	108 (35.3)	27 (30.7)	45 (35.2)	36 (36.4)	15 (10.1)	1 (5.9)	10 (13.7)	4 (6.2)
Staphylococcus aureus	17 (5.6)	3 (3.4)	7 (5.5)	8 (8.1)	14 (9.4)	0 (0)	7 (9.6)	7 (10.8)
Streptococcus spp.	19 (6.2)	5 (5.7)	6 (4.7)	8 (8.1)	7 (4.7)	0 (0)	4 (5.5)	3 (4.6)
Streptococcus viridans	1 (0.3)	0 (0)	0 (0)	1 (1.0)	3 (2.0)	0 (0)	2 (2.7)	1 (1.5)
Enterococcus faecalis	0 (0)	0 (0)	0 (0)	0 (0)	3 (2.0)	0 (0)	0 (0)	3 (4.6)
Streptococcus agalactiae	2 (0.7)	1 (1.1)	1 (0.8)	0 (0)	1 (0.7)	0 (0)	1 (1.4)	0 (0)
Streptococcus pyogenes	2 (0.7)	0 (0)	0 (0)	2 (2.0)	0 (0)	0 (0)	0 (0)	0 (0)
Enterococcus faecium	1 (0.3)	0 (0)	0 (0)	1 (1.0)	0 (0)	0 (0)	0 (0)	0 (0)
Enterococcus spp.	0 (0)	0 (0)	0 (0)	0 (0)	0 (0)	0 (0)	0 (0)	1 (1.5)
**Virus**	**72 (23.5)**	**19 (21.6)**	**32 (25.0)**	**23 (23.2)**	**73 (49.0)**	**10 (58.8)**	**36 (49.3)**	**27 (41.5)**
Cytomegalovirus	28 (9.2)	4 (4.5)	15 (11.7)	9 (9.1)	26 (17.4)	2 (11.8)	11 (15.1)	13 (20.0)
Respiratory syncytial virus	15 (4.9)	7 (8.0)	6 (4.7)	2 (2.0)	14 (9.4)	3 (17.6)	7 (9.6)	4 (6.2)
Adenovirus	6 (2.0)	0 (0)	2 (1.6)	4 (4.0)	19 (12.8)	5 (29.4)	8 (11.0)	6 (9.2)
Influenza A	7 (2.3)	2 (2.3)	4 (3.1)	1 (1.0)	3 (2.0)	0 (0)	1 (1.4)	2 (3.1)
Parainfluenza virus type 3	2 (0.7)	0 (0)	0 (0)	2 (2.0)	8 (5.4)	0 (0)	6 (8.2)	2 (3.1)
Rhinovirus	4 (1.3)	3 (3.4)	1 (0.8)	0 (0)	6 (4.0)	0 (0)	2 (2.7)	4 (6.2)
Human metapneumovirus	4 (1.3)	3 (3.4)	0 (0)	1 (1.0)	3 (2.0)	0 (0)	3 (4.1)	0 (0)
Influenza B	4 (1.3)	1 (1.1)	2 (1.6)	2 (2.0)	2 (1.3)	1 (5.9)	0 (0)	1 (1.5)
Parainfluenza virus type 1	3 (1.0)	1 (1.1)	0 (0)	2 (2.0)	2 (1.3)	0 (0)	2 (2.7)	0 (0)
Parainfluenza virus type 4	2 (0.7)	0 (0)	1 (0.8)	1 (1.0)	1 (0.7)	0 (0)	0 (0)	1 (1.5)
Human coronavirus OC43	0 (0)	0 (0)	0 (0)	0 (0)	1 (0.7)	0 (0)	0 (0)	1 (1.5)
Parainfluenza virus type 2	1 (0.3)	0 (0)	1 (0.8)	0 (0)	0 (0)	0 (0)	0 (0)	0 (0)
SARS-CoV-2	1 (0.3)	0 (0)	1 (0.8)	0 (0)	0 (0)	0 (0)	0 (0)	0 (0)

**Fungus**	**24 (7.8)**	**4 (4.5)**	**11 (8.6)**	**9 (9.1)**	**11 (7.4)**	**2 (11.8)**	**7 (9.6)**	**2 (3.1)**
Pneumocystis jirovecii	19 (6.2)	4 (4.5)	9 (7.0)	6 (6.1)	8 (5.4)	2 (11.8)	6 (8.2)	0 (0)
Candida albicans	3 (1.0)	0 (0)	1 (0.8)	2 (2.0)	0 (0)	0 (0)	0 (0)	0 (0)
Candida spp.	2 (0.7)	0 (0)	1 (0.8)	1 (1.0)	1 (0.7)	0 (0)	1 (1.4)	0 (0)
Candida auris	0 (0)	0 (0)	0 (0)	0 (0)	1 (0.7)	0 (0)	0 (0)	1 (1.5)
Pneumocystis spp.	0 (0)	0 (0)	0 (0)	0 (0)	1 (0.7)	0 (0)	0 (0)	1 (1.5)
**No pathogen implicated**	63 (20·6)	26 (29·5)	27 (21.1)	10 (10.1)	15 (10·1)	1 (5·9)	9 (12.3)	5 (7.7)

[a] Several deaths had pneumonia as both underlying and immediate/antecedent cause of death, so the number of underlying plus immediate/antecedent exceeds the total number of deaths.

[b] Other than *Klebsiella pneumoniae*.

Supplemental Table S10. Pathogens identified as causing pneumonia deaths stratified by age group and by whether the pneumonia death 1) occurred in the community or with fewer than 120 hours in the hospital or 2) occurred 120 or more hours after hospital admission[a], CHAMPS Network, December 16th 2016 to December 31st, 2022.

	Community deaths or <120 hours of hospital stay				Hospital-associated (i.e., deaths occurring ≥120 hours after admission to hospital)			
	All	1-6 months	6-12 months	12-59 months	All	1-6 months	6-12 months	12-59 months
	N = 344	N = 114	N = 72	N = 158	N = 111	N = 56	N = 22	N = 33
**Pathogen**	n (%)	n (%)	n (%)	n (%)	n (%)	n (%)	n (%)	n (%)
**Gram negative bacteria**	**167 (48·5)**	**60 (52·6)**	**30 (41·7)**	**77 (48·7)**	**65 (58·6)**	**36 (64·3)**	**10 (45·5)**	**19 (57·6)**
Klebsiella pneumoniae	94 (27·3)	38 (33·3)	17 (23·6)	39 (24·7)	48 (43·2)	25 (44·6)	6 (27·3)	17 (51·5)
Non-typeable *Haemophilus influenzae*	39 (11·3)	9 (7·9)	7 (9·7)	23 (14·6)	6 (5·4)	2 (3·6)	2 (9·1)	2 (6·1)
Acinetobacter baumannii	12 (3·5)	9 (7·9)	1 (1·4)	2 (1·3)	18 (16·2)	11 (19·6)	3 (13·6)	4 (12·1)
Pseudomonas aeruginosa	11 (3·2)	3 (2·6)	1 (1·4)	7 (4·4)	14 (12·6)	5 (8·9)	3 (13·6)	6 (18·2)
Escherichia coli	14 (4·1)	5 (4·4)	3 (4·2)	6 (3·8)	2 (1·8)	2 (3·6)	0 (0)	0 (0)
Haemophilus influenzae Type A	15 (4·4)	3 (2·6)	2 (2·8)	10 (6·3)	1 (0·9)	0 (0)	1 (4·5)	0 (0)
Moraxella catarrhalis	13 (3·8)	4 (3·5)	3 (4·2)	6 (3·8)	0 (0)	0 (0)	0 (0)	0 (0)
Bordetella pertussis	4 (1·2)	4 (3·5)	0 (0)	0 (0)	3 (2·7)	3 (5·4)	0 (0)	0 (0)
Haemophilus influenzae Type B	3 (0·9)	2 (1·8)	1 (1·4)	0 (0)	0 (0)	0 (0)	0 (0)	0 (0)
Klebsiella spp·[a]	2 (0·6)	0 (0)	1 (1·4)	1 (0·6)	1 (0·9)	0 (0)	1 (4·5)	0 (0)
Salmonella spp·	3 (0·9)	0 (0)	1 (1·4)	2 (1·3)	0 (0)	0 (0)	0 (0)	0 (0)
Other	7 (2·0)	3 (2·6)	1 (1·4)	3 (1·9)	2 (1·8)	2 (3·6)	0 (0)	0 (0)
**Gram positive bacteria**	**156 (45·3)**	**47 (41·2)**	**39 (54·2)**	**70 (44·3)**	**26 (23·4)**	**14 (25·0)**	**2 (9·1)**	**10 (30·3)**
Streptococcus pneumoniae	113 (32·8)	29 (25·4)	31 (43·1)	53 (33·5)	10 (9·0)	4 (7·1)	2 (9·1)	4 (12·1)
Staphylococcus aureus	23 (6·7)	9 (7·9)	4 (5·6)	10 (6·3)	8 (7·2)	7 (12·5)	0 (0)	1 (3·0)
Streptococcus spp·	22 (6·4)	8 (7·0)	5 (6·9)	9 (5·7)	4 (3·6)	1 (1·8)	0 (0)	3 (9·1)
Streptococcus viridans	1 (0·3)	0 (0)	1 (1·4)	0 (0)	3 (2·7)	2 (3·6)	0 (0)	1 (3·0)
Enterococcus faecalis	0 (0)	0 (0)	0 (0)	0 (0)	3 (2·7)	1 (1·8)	0 (0)	2 (6·1)
Streptococcus agalactiae	3 (0·9)	1 (0·9)	0 (0)	2 (1·3)	0 (0)	0 (0)	0 (0)	0 (0)
Other	3 (0·9)	3 (2·6)	0 (0)	0 (0)	1 (0·9)	0 (0)	0 (0)	1 (3·0)
**Virus**	**92 (26·7)**	**36 (31·6)**	**22 (30·6)**	**34 (21·5)**	**53 (47·7)**	**31 (55·4)**	**10 (45·5)**	**12 (36·4)**
Cytomegalovirus	32 (9·3)	16 (14·0)	8 (11·1)	8 (5·1)	22 (19·8)	13 (23·2)	5 (22·7)	4 (12·1)
Respiratory syncytial virus	22 (6·4)	11 (9·6)	4 (5·6)	7 (4·4)	7 (6·3)	6 (10·7)	0 (0)	1 (3·0)
Adenovirus	11 (3·2)	3 (2·6)	2 (2·8)	6 (3·8)	14 (12·6)	7 (12·5)	4 (18·2)	3 (9·1)
Influenza A	9 (2·6)	0 (0)	3 (4·2)	6 (3·8)	1 (0·9)	1 (1·8)	0 (0)	0 (0)
Parainfluenza virus type 3	4 (1·2)	0 (0)	2 (2·8)	2 (1·3)	6 (5·4)	5 (8·9)	0 (0)	1 (3·0)
Rhinovirus	5 (1·5)	2 (1·8)	1 (1·4)	2 (1·3)	5 (4·5)	4 (7·1)	0 (0)	1 (3·0)
Human metapneumovirus	6 (1·7)	3 (2·6)	1 (1·4)	2 (1·3)	1 (0·9)	0 (0)	0 (0)	1 (3·0)
Influenza B	4 (1·2)	2 (1·8)	1 (1·4)	1 (0·6)	2 (1·8)	0 (0)	1 (4·5)	1 (3·0)
Parainfluenza virus type 1	3 (0·9)	0 (0)	2 (2·8)	1 (0·6)	2 (1·8)	0 (0)	1 (4·5)	1 (3·0)
Parainfluenza virus type 4	2 (0·6)	0 (0)	1 (1·4)	1 (0·6)	1 (0·9)	0 (0)	1 (4·5)	0 (0)
Other	1 (0·3)	1 (0·9)	0 (0)	0 (0)	1 (0·9)	1 (1·8)	0 (0)	0 (0)
**Fungus**	**27 (7·8)**	**16 (14·0)**	**3 (4·2)**	**8 (5·1)**	**8 (7·2)**	**6 (10·7)**	**2 (9·1)**	**0 (0)**
Pneumocystis jirovecii	20 (5·8)	13 (11·4)	2 (2·8)	5 (3·2)	7 (6·3)	6 (10·7)	1 (4·5)	0 (0)
Candida albicans	3 (0·9)	0 (0)	0 (0)	3 (1·9)	0 (0)	0 (0)	0 (0)	0 (0)
Candida spp·	3 (0·9)	2 (1·8)	1 (1·4)	0 (0)	0 (0)	0 (0)	0 (0)	0 (0)
Other	1 (0·3)	1 (0·9)	0 (0)	0 (0)	1 (0·9)	0 (0)	1 (4·5)	0 (0)
**No pathogen implicated**	68 (19·8)	21 (18·4)	13 (18·1)	34 (21·5)	10 (9·0)	1 (1·8)	4 (18·2)	5 (15·2)
**Number of cases with only 1 pathogen implicated**	117 (34·0)	33 (28·9)	26 (36·1)	58 (36·7)	42 (37·8)	21 (37·5)	8 (36·4)	13 (39·4)
**Number of cases with 2 pathogens implicated**	85 (24·7)	29 (25·4)	18 (25·0)	38 (24·1)	30 (27·0)	19 (33·9)	6 (27·3)	5 (15·2)

**Number of cases with 3 pathogens implicated**	74 (21·5)	31 (27·2)	15 (20·8)	28 (17·7)	25 (22·5)	12 (21·4)	4 (18·2)	9 (27·3)
**Number of cases with 4 or more pathogen implicated**	0 (0)	0 (0)	0 (0)	0 (0)	4 (3·6)	3 (5·4)	0 (0)	1 (3·0)
**Median number of pathogens implicated per case[b]**	2 (1, 3)	2 (1, 3)	2 (1, 3)	2 (1, 2)	2 (1, 3)	2 (1, 3)	2 (1, 2)	2 (1, 3)
[b] Other than *Klebsiella pneumoniae*.								
[c] Among deaths with at least one pathogen implicated.								

**Supplemental Table 11**: CHAMPS site specific analysis of pathogens attributed to causing pneumonia deaths which occurred in the community or within 72 hours of admission; CHAMPS Network, December 16th 2016 to December 31st, 2022							
	South Africa (N = 61)	Kenya (N = 66)	Mozambique (N = 68)	Ethiopia (N = 19)	Sierra Leone (N = 63)	Mali (N = 28)	Bangladesh (N = 1)
**Pathogen**							
**Gram negative bacteria**	**33 (54.1)**	**23 (34.8)**	**41 (60.3)**	**11 (57.9)**	**25 (39.7)**	**15 (53.6)**	**0 (0)**
Klebsiella pneumoniae	19 (31.1)	16 (24.2)	12 (17.6)	9 (47.4)	16 (25.4)	6 (21.4)	0 (0)
Non-typeable *Haemophilus influenzae*	10 (16.4)	5 (7.6)	9 (13.2)	3 (15.8)	3 (4.8)	7 (25.0)	0 (0)
Acinetobacter baumannii	4 (6.6)	0 (0)	0 (0)	2 (10.5)	5 (7.9)	0 (0)	0 (0)
Pseudomonas aeruginosa	2 (3.3)	1 (1.5)	3 (4.4)	2 (10.5)	1 (1.6)	1 (3.6)	0 (0)
Escherichia coli	5 (8.2)	2 (3.0)	5 (7.4)	0 (0)	1 (1.6)	1 (3.6)	0 (0)
Haemophilus influenzae Type A	0 (0)	1 (1.5)	12 (17.6)	0 (0)	1 (1.6)	0 (0)	0 (0)
Moraxella catarrhalis	2 (3.3)	0 (0)	9 (13.2)	0 (0)	0 (0)	1 (3.6)	0 (0)
Bordetella pertussis	1 (1.6)	1 (1.5)	1 (1.5)	0 (0)	0 (0)	1 (3.6)	0 (0)
Haemophilus influenzae Type B	1 (1.6)	0 (0)	1 (1.5)	0 (0)	0 (0)	1 (3.6)	0 (0)
Klebsiella spp.	0 (0)	0 (0)	1 (1.5)	0 (0)	0 (0)	1 (3.6)	0 (0)
Salmonella spp.	0 (0)	0 (0)	0 (0)	0 (0)	3 (4.8)	0 (0)	0 (0)
Bordetella spp.	0 (0)	0 (0)	1 (1.5)	0 (0)	1 (1.6)	0 (0)	0 (0)
Haemophilus parainfluenzae	0 (0)	1 (1.5)	1 (1.5)	0 (0)	0 (0)	0 (0)	0 (0)
Haemophilus spp.	0 (0)	0 (0)	2 (2.9)	0 (0)	0 (0)	0 (0)	0 (0)
Chlamydia trachomatis	1 (1.6)	0 (0)	0 (0)	0 (0)	0 (0)	0 (0)	0 (0)
**Gram positive bacteria**	**28 (45.9)**	**31 (47.0)**	**40 (58.8)**	**9 (47.4)**	**18 (28.6)**	**16 (57.1)**	**0 (0)**
Streptococcus pneumoniae	16 (26.2)	21 (31.8)	36 (52.9)	9 (47.4)	12 (19.0)	14 (50.0)	0 (0)
Staphylococcus aureus	8 (13.1)	5 (7.6)	1 (1.5)	0 (0)	2 (3.2)	1 (3.6)	0 (0)
Streptococcus spp.	4 (6.6)	7 (10.6)	3 (4.4)	0 (0)	0 (0)	0 (0)	0 (0)
Streptococcus viridans	1 (1.6)	0 (0)	0 (0)	0 (0)	0 (0)	0 (0)	0 (0)
Enterococcus faecalis	0 (0)	0 (0)	0 (0)	0 (0)	0 (0)	0 (0)	0 (0)
Streptococcus agalactiae	0 (0)	0 (0)	1 (1.5)	0 (0)	1 (1.6)	0 (0)	0 (0)
Streptococcus pyogenes	1 (1.6)	0 (0)	0 (0)	1 (5.3)	0 (0)	0 (0)	0 (0)
Enterococcus faecium	1 (1.6)	0 (0)	0 (0)	0 (0)	0 (0)	0 (0)	0 (0)
Enterococcus spp.	0 (0)	0 (0)	0 (0)	0 (0)	3 (4.8)	2 (7.1)	0 (0)
**Virus**	**17 (27.9)**	**22 (33.3)**	**9 (13.2)**	**3 (15.8)**	**11 (17.5)**	**10 (35.7)**	**0 (0)**
Cytomegalovirus	9 (14.8)	12 (18.2)	2 (2.9)	0 (0)	4 (6.3)	1 (3.6)	0 (0)
Respiratory syncytial virus	3 (4.9)	3 (4.5)	3 (4.4)	1 (5.3)	3 (4.8)	2 (7.1)	0 (0)
Adenovirus	2 (3.3)	1 (1.5)	0 (0)	0 (0)	2 (3.2)	1 (3.6)	0 (0)
Influenza A	0 (0)	2 (3.0)	1 (1.5)	0 (0)	2 (3.2)	2 (7.1)	0 (0)
Parainfluenza virus type 3	1 (1.6)	1 (1.5)	0 (0)	0 (0)	0 (0)	0 (0)	0 (0)
Rhinovirus	3 (4.9)	0 (0)	0 (0)	1 (5.3)	0 (0)	0 (0)	0 (0)
Human metapneumovirus	1 (1.6)	0 (0)	1 (1.5)	0 (0)	1 (1.6)	1 (3.6)	0 (0)
Influenza B	0 (0)	1 (1.5)	2 (2.9)	1 (5.3)	0 (0)	0 (0)	0 (0)
Parainfluenza virus type 1	0 (0)	1 (1.5)	0 (0)	0 (0)	0 (0)	2 (7.1)	0 (0)
Parainfluenza virus type 4	0 (0)	1 (1.5)	0 (0)	0 (0)	0 (0)	1 (3.6)	0 (0)
Human coronavirus OC43	0 (0)	0 (0)	0 (0)	0 (0)	0 (0)	0 (0)	0 (0)
Parainfluenza virus type 2	0 (0)	1 (1.5)	0 (0)	0 (0)	0 (0)	0 (0)	0 (0)
SARS-CoV-2	0 (0)	0 (0)	1 (1.5)	0 (0)	0 (0)	0 (0)	0 (0)
**Fungus**	**9 (14.8)**	**5 (7.6)**	**2 (2.9)**	**2 (10.5)**	**5 (7.9)**	**1 (3.6)**	**0 (0)**
Pneumocystis jirovecii	7 (11.5)	5 (7.6)	1 (1.5)	2 (10.5)	3 (4.8)	1 (3.6)	0 (0)
Candida albicans	1 (1.6)	0 (0)	1 (1.5)	0 (0)	1 (1.6)	0 (0)	0 (0)
Candida spp.	1 (1.6)	0 (0)	0 (0)	0 (0)	1 (1.6)	0 (0)	0 (0)
**No pathogen**	**11 (18.0)**	**15 (22.7)**	**15 (22.1)**	**3 (15.8)**	**16 (25.4)**	**2 (7.1)**	**1 (100)**

Supplemental Table S12: CHAMPS site specific analysis of pathogens attributed to causing overall pneumonia associated deaths; CHAMPS Network, December 16th 2016 to December 31st, 2022.

	South Africa (N = 151)	Kenya (N = 78)	Mozambique (N = 90)	Ethiopia (N = 20)	Sierra Leone (N = 84)	Mali (N = 30)	Bangladesh (N = 2)
**Pathogen**							
**Gram negative bacteria**	**87 (57.6)**	**29 (37.2)**	**53 (58.9)**	**12 (60.0)**	**36 (42.9)**	**15 (50.0)**	**0 (0)**
Klebsiella pneumoniae	56 (37.1)	20 (25.6)	24 (26.7)	10 (50.0)	26 (31.0)	6 (20.0)	0 (0)
Non-typeable *Haemophilus influenzae*	15 (9.9)	6 (7.7)	9 (10.0)	3 (15.0)	5 (6.0)	7 (23.3)	0 (0)
Acinetobacter baumannii	20 (13.2)	0 (0)	0 (0)	3 (15.0)	7 (8.3)	0 (0)	0 (0)
Pseudomonas aeruginosa	11 (7.3)	1 (1.3)	8 (8.9)	2 (10.0)	2 (2.4)	1 (3.3)	0 (0)
Escherichia coli	6 (4.0)	2 (2.6)	6 (6.7)	0 (0)	1 (1.2)	1 (3.3)	0 (0)
Haemophilus influenzae Type A	0 (0)	2 (2.6)	13 (14.4)	0 (0)	1 (1.2)	0 (0)	0 (0)
Moraxella catarrhalis	3 (2.0)	0 (0)	9 (10.0)	0 (0)	0 (0)	1 (3.3)	0 (0)
Bordetella pertussis	3 (2.0)	1 (1.3)	1 (1.1)	0 (0)	1 (1.2)	1 (3.3)	0 (0)
Haemophilus influenzae Type B	1 (0.7)	0 (0)	1 (1.1)	0 (0)	0 (0)	1 (3.3)	0 (0)
Klebsiella spp.	0 (0)	1 (1.3)	1 (1.1)	0 (0)	0 (0)	1 (3.3)	0 (0)
Salmonella spp.	0 (0)	0 (0)	0 (0)	0 (0)	3 (3.6)	0 (0)	0 (0)
Bordetella spp.	0 (0)	0 (0)	1 (1.1)	0 (0)	1 (1.2)	0 (0)	0 (0)
Haemophilus parainfluenzae	0 (0)	1 (1.3)	1 (1.1)	0 (0)	0 (0)	0 (0)	0 (0)
Haemophilus spp.	0 (0)	0 (0)	2 (2.2)	0 (0)	0 (0)	0 (0)	0 (0)
Chlamydia trachomatis	1 (0.7)	0 (0)	0 (0)	0 (0)	0 (0)	0 (0)	0 (0)
Haemophilus aphrophilus	1 (0.7)	0 (0)	0 (0)	0 (0)	0 (0)	0 (0)	0 (0)
Ureaplasma spp.	1 (0.7)	0 (0)	0 (0)	0 (0)	0 (0)	0 (0)	0 (0)
**Gram positive bacteria**	**56 (37.1)**	**33 (42.3)**	**45 (50.0)**	**9 (45.0)**	**22 (26.2)**	**17 (56.7)**	**0 (0)**
Streptococcus pneumoniae	23 (15.2)	22 (28.2)	38 (42.2)	9 (45.0)	16 (19.0)	15 (50.0)	0 (0)
Staphylococcus aureus	22 (14.6)	5 (6.4)	1 (1.1)	0 (0)	2 (2.4)	1 (3.3)	0 (0)
Streptococcus spp.	7 (4.6)	8 (10.3)	6 (6.7)	0 (0)	3 (3.6)	2 (6.7)	0 (0)
Streptococcus viridans	4 (2.6)	0 (0)	0 (0)	0 (0)	0 (0)	0 (0)	0 (0)
Enterococcus faecalis	3 (2.0)	0 (0)	0 (0)	0 (0)	0 (0)	0 (0)	0 (0)
Streptococcus agalactiae	1 (0.7)	0 (0)	1 (1.1)	0 (0)	1 (1.2)	0 (0)	0 (0)
Streptococcus pyogenes	1 (0.7)	0 (0)	0 (0)	1 (5.0)	0 (0)	0 (0)	0 (0)
Enterococcus faecium	1 (0.7)	0 (0)	0 (0)	0 (0)	0 (0)	0 (0)	0 (0)
Enterococcus spp.	1 (0.7)	0 (0)	0 (0)	0 (0)	0 (0)	0 (0)	0 (0)
**Virus**	**69 (45.7)**	**27 (34.6)**	**18 (20.0)**	**3 (15.0)**	**17 (20.2)**	**11 (36.7)**	**0 (0)**
Cytomegalovirus	26 (17.2)	13 (16.7)	7 (7.8)	0 (0)	7 (8.3)	1 (3.3)	0 (0)
Respiratory syncytial virus	15 (9.9)	5 (6.4)	3 (3.3)	1 (5.0)	3 (3.6)	2 (6.7)	0 (0)
Adenovirus	14 (9.3)	2 (2.6)	3 (3.3)	0 (0)	4 (4.8)	2 (6.7)	0 (0)
Influenza A	2 (1.3)	2 (2.6)	1 (1.1)	0 (0)	3 (3.6)	2 (6.7)	0 (0)
Parainfluenza virus type 3	9 (6.0)	1 (1.3)	0 (0)	0 (0)	0 (0)	0 (0)	0 (0)
Rhinovirus	8 (5.3)	0 (0)	1 (1.1)	1 (5.0)	0 (0)	0 (0)	0 (0)
Human metapneumovirus	2 (1.3)	1 (1.3)	1 (1.1)	0 (0)	2 (2.4)	1 (3.3)	0 (0)
Influenza B	0 (0)	2 (2.6)	3 (3.3)	1 (5.0)	0 (0)	0 (0)	0 (0)
Parainfluenza virus type 1	1 (0.7)	2 (2.6)	0 (0)	0 (0)	0 (0)	2 (6.7)	0 (0)
Parainfluenza virus type 4	0 (0)	1 (1.3)	0 (0)	0 (0)	1 (1.2)	1 (3.3)	0 (0)
Human coronavirus OC43	1 (0.7)	0 (0)	0 (0)	0 (0)	0 (0)	0 (0)	0 (0)
Parainfluenza virus type 2	0 (0)	1 (1.3)	0 (0)	0 (0)	0 (0)	0 (0)	0 (0)
SARS-CoV-2	0 (0)	0 (0)	1 (1.1)	0 (0)	0 (0)	0 (0)	0 (0)
**Fungus**	**19 (12.6)**	**5 (6.4)**	**2 (2.2)**	**2 (10.0)**	**6 (7.1)**	**1 (3.3)**	**0 (0)**
Pneumocystis jirovecii	14 (9.3)	5 (6.4)	1 (1.1)	2 (10.0)	4 (4.8)	1 (3.3)	0 (0)
Candida albicans	1 (0.7)	0 (0)	1 (1.1)	0 (0)	1 (1.2)	0 (0)	0 (0)
Candida spp.	2 (1.3)	0 (0)	0 (0)	0 (0)	1 (1.2)	0 (0)	0 (0)
Candida auris	1 (0.7)	0 (0)	0 (0)	0 (0)	0 (0)	0 (0)	0 (0)

Pneumocystis spp.	1 (0.7)	0 (0)	0 (0)	0 (0)	0 (0)	0 (0)	0 (0)
**No pathogen**	**11 (18.0)**	**15 (22.7)**	**15 (22.1)**	**3 (15.0)**	**18 (21.4)**	**2 (6.7)**	**2 (100)**

Supplemental Table S13: CHAMPS site specific analysis of pathogens attributed to causing pneumonia deaths which occurred 72 hours or later after hospital admission; CHAMPS Network, December 16th 2016 to December 31st, 2022.

	South Africa (N = 90)	Kenya (N = 12)	Mozambique (N = 22)	Ethiopia (N = 1)	Sierra Leone (N = 21)	Mali (N = 2)	Bangladesh (N = 1)
**Pathogen**							
**Gram negative bacteria**	**54 (60.0)**	**6 (50.0)**	**12 (54.5)**	**1 (100.0)**	**11 (52.4)**	**0 (0)**	**0 (0)**
Klebsiella pneumoniae	37 (41.1)	4 (33.3)	12 (54.5)	1 (100.0)	10 (47.6)	0 (0)	0 (0)
Non-typeable *Haemophilus influenzae*	5 (5.6)	1 (8.3)	0 (0)	0 (0)	2 (9.5)	0 (0)	0 (0)
Acinetobacter baumannii	16 (17.8)	0 (0)	0 (0)	1 (100.0)	2 (9.5)	0 (0)	0 (0)
Pseudomonas aeruginosa	9 (10.0)	0 (0)	5 (22.7)	0 (0)	1 (4.8)	0 (0)	0 (0)
Escherichia coli	1 (1.1)	0 (0)	1 (4.5)	0 (0)	0 (0)	0 (0)	0 (0)
Haemophilus influenzae Type A	0 (0)	1 (8.3)	1 (4.5)	0 (0)	0 (0)	0 (0)	0 (0)
Moraxella catarrhalis	1 (1.1)	0 (0)	0 (0)	0 (0)	0 (0)	0 (0)	0 (0)
Bordetella pertussis	2 (2.2)	0 (0)	0 (0)	0 (0)	1 (4.8)	0 (0)	0 (0)
Klebsiella spp.	0 (0)	1 (8.3)	0 (0)	0 (0)	0 (0)	0 (0)	0 (0)
Haemophilus aphrophilus	1 (1.1)	0 (0)	0 (0)	0 (0)	0 (0)	0 (0)	0 (0)
Ureaplasma spp.	1 (1.1)	0 (0)	0 (0)	0 (0)	0 (0)	0 (0)	0 (0)
**Gram positive bacteria**	**28 (31.1)**	**2 (16.7)**	**5 (22.7)**	**0 (0)**	**4 (19.0)**	**1 (50.0)**	**0 (0)**
Streptococcus pneumoniae	7 (7.8)	1 (8.3)	2 (9.1)	0 (0)	4 (19.0)	1 (50.0)	0 (0)
Staphylococcus aureus	14 (15.6)	0 (0)	0 (0)	0 (0)	0 (0)	0 (0)	0 (0)
Streptococcus spp.	3 (3.3)	1 (8.3)	3 (13.6)	0 (0)	0 (0)	0 (0)	0 (0)
Streptococcus viridans	3 (3.3)	0 (0)	0 (0)	0 (0)	0 (0)	0 (0)	0 (0)
Enterococcus faecalis	3 (3.3)	0 (0)	0 (0)	0 (0)	0 (0)	0 (0)	0 (0)
Streptococcus agalactiae	1 (1.1)	0 (0)	0 (0)	0 (0)	0 (0)	0 (0)	0 (0)
Enterococcus spp.	1 (1.1)	0 (0)	0 (0)	0 (0)	0 (0)	0 (0)	0 (0)
**Virus**	**52 (57.8)**	**5 (41.7)**	**9 (40.9)**	**0 (0)**	**6 (28.6)**	**1 (50.0)**	**0 (0)**
Cytomegalovirus	17 (18.9)	1 (8.3)	5 (22.7)	0 (0)	3 (14.3)	0 (0)	0 (0)
Respiratory syncytial virus	12 (13.3)	2 (16.7)	0 (0)	0 (0)	0 (0)	0 (0)	0 (0)
Adenovirus	12 (13.3)	1 (8.3)	3 (13.6)	0 (0)	2 (9.5)	1 (50.0)	0 (0)
Influenza A	2 (2.2)	0 (0)	0 (0)	0 (0)	1 (4.8)	0 (0)	0 (0)
Parainfluenza virus type 3	8 (8.9)	0 (0)	0 (0)	0 (0)	0 (0)	0 (0)	0 (0)
Rhinovirus	5 (5.6)	0 (0)	1 (4.5)	0 (0)	0 (0)	0 (0)	0 (0)
Human metapneumovirus	1 (1.1)	1 (8.3)	0 (0)	0 (0)	1 (4.8)	0 (0)	0 (0)
Influenza B	0 (0)	1 (8.3)	1 (4.5)	0 (0)	0 (0)	0 (0)	0 (0)
Parainfluenza virus type 1	1 (1.1)	1 (8.3)	0 (0)	0 (0)	0 (0)	0 (0)	0 (0)
Parainfluenza virus type 4	0 (0)	0 (0)	0 (0)	0 (0)	1 (4.8)	0 (0)	0 (0)
Human coronavirus OC43	1 (1.1)	0 (0)	0 (0)	0 (0)	0 (0)	0 (0)	0 (0)
**Fungus**	**10 (11.1)**	**0 (0)**	**0 (0)**	**0 (0)**	**1 (4.8)**	**0 (0)**	**0 (0)**
Pneumocystis jirovecii	7 (7.8)	0 (0)	0 (0)	0 (0)	1 (4.8)	0 (0)	0 (0)
Candida spp.	1 (1.1)	0 (0)	0 (0)	0 (0)	0 (0)	0 (0)	0 (0)
Candida auris	1 (1.1)	0 (0)	0 (0)	0 (0)	0 (0)	0 (0)	0 (0)
Pneumocystis spp.	1 (1.1)	0 (0)	0 (0)	0 (0)	0 (0)	0 (0)	0 (0)
**No pathogen**	**4 (4.4)**	**2 (16.7)**	**6 (27.3)**	**0 (0)**	**2 (9.5)**	**0 (0)**	**100 (1)**

Supplemental Table S14: Cycle threshold values for cytomegalovirus (CMV) on the TacMan Array Card nucleic acid amplification assay in children who tested positive for CMV, stratified by whether or not CMV was attributed in the etiology of pneumonia-associated death or not implicated in the causal pathway.

Sample source	Pneumonia attributed to CMV in the causal chain (N=54)							CMV in decedents where it was included in the causal pathway to death (N=391[a])						
	Positive	Min	Lower quantile	Median	Mean	Upper quantile	Max	Positive	Min	Lower quantile	Median	Mean	Upper quantile	Max
Tissue specimen from lung	51	18.65	24.09	25.93	26.7	29.08	39.24	229	18.80	29.13	31.20	31.08	33.63	40.57
Nasopharyngeal swab	40	15.8	22.93	25.78	26.21	29.56	33.75	319	16.83	26.13	29.59	29.22	32.40	42.47
Whole blood	15	18	26.9	29.99	29.65	32.74	39.24	221	20.27	31.34	32.72	32.41	34.91	40.47
Cerebrospinal fluid sample	6	29.31	33.37	34.26	33.54	34.8	35.38	30	27.40	31.17	32.97	33.01	35.46	37.92

[a] Of the 455 pneumonia deaths, 64 (14.1%) had CMV anywhere in the causal chain. Of the 64, 54 had CMV implicated in pneumonia. Therefore, there were 10 pneumonia deaths that had CMV in the causal chain that was not implicated the pneumonia, which were excluded from this table.

Supplemental Figure 1: Inclusion and exclusion criteria for minimally invasive tissue sampling (MITS) and non-MITS enrollment (ref *Clin Infect Dis*, Volume 69, Issue Supplement_4, 15 October 2019, Pages S262–S273, https://doi.org/10.1093/cid/ciz599

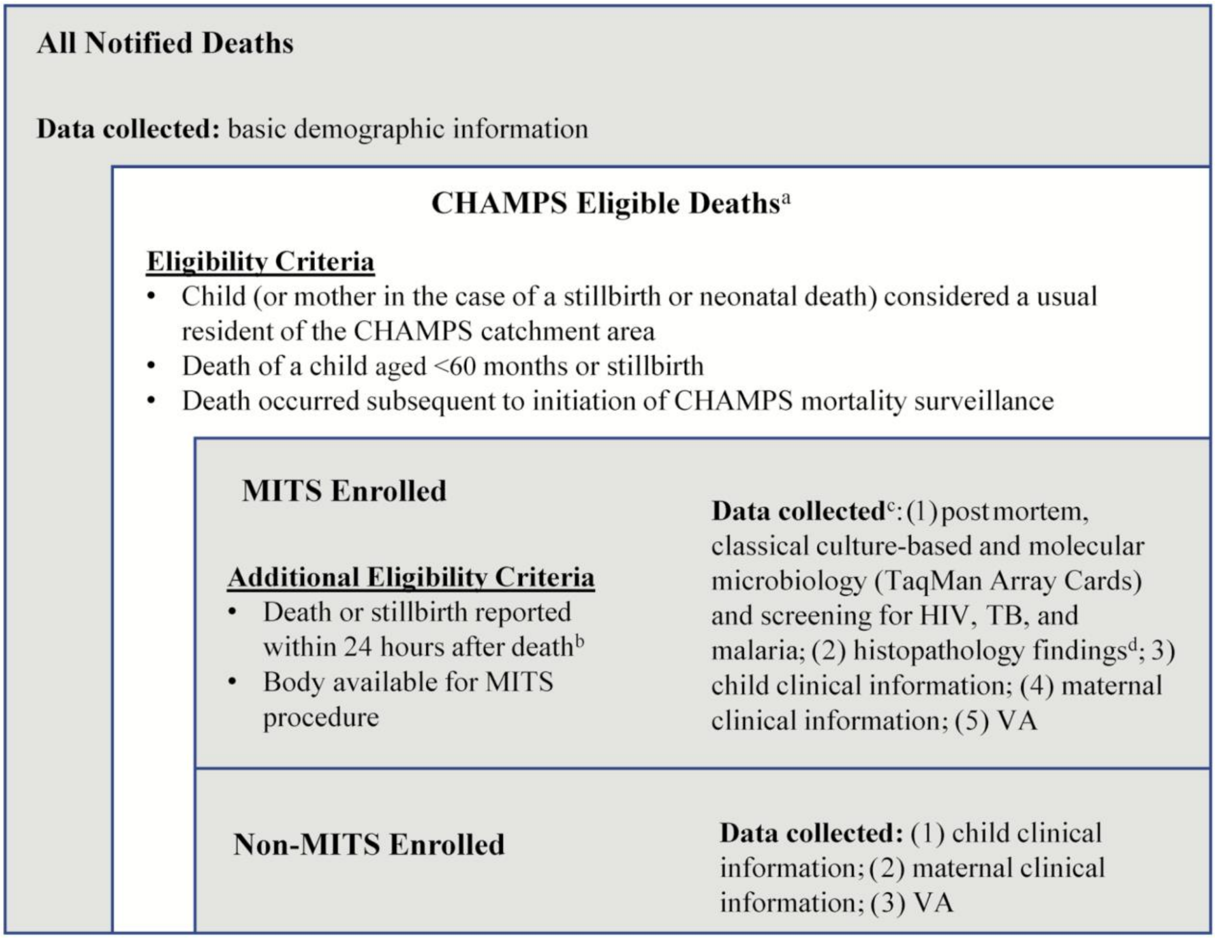

[a]A small proportion of confirmed Child Health and Mortality Prevention Surveillance (CHAMPS) eligible deaths (ie, family was approached for eligibility screening and confirmed eligibility information) are not enrolled in CHAMPS due to parental nonconsent or loss to follow-up. [b]The MITS timeframe may be extended up to 72 hours after death if body is refrigerated shortly after death. [c]Circumstances may prevent the MITS from being conducted after MITS consent has been obtained. In these infrequent cases, data collection aligns with non-MITS procedures. [d]Histology is conducted at the site and at the central pathology laboratory located at the US Centers for Disease Control and Prevention. Abbreviations: CHAMPS, Child Health and Mortality Prevention Surveillance; HIV, human immunodeficiency virus; MITS, minimally invasive tissue sampling; TB, tuberculosis; VA, verbal autopsy.

Supplemental Figure S2. Flowchart of enrolled under-five infant and child deaths from CHAMPS sites from 2016 to 2022 that had minimally invasive tissue samples (MITS) and consent only for verbal autopsy and clinical abstraction (Non-MITS) and included in the analysis.

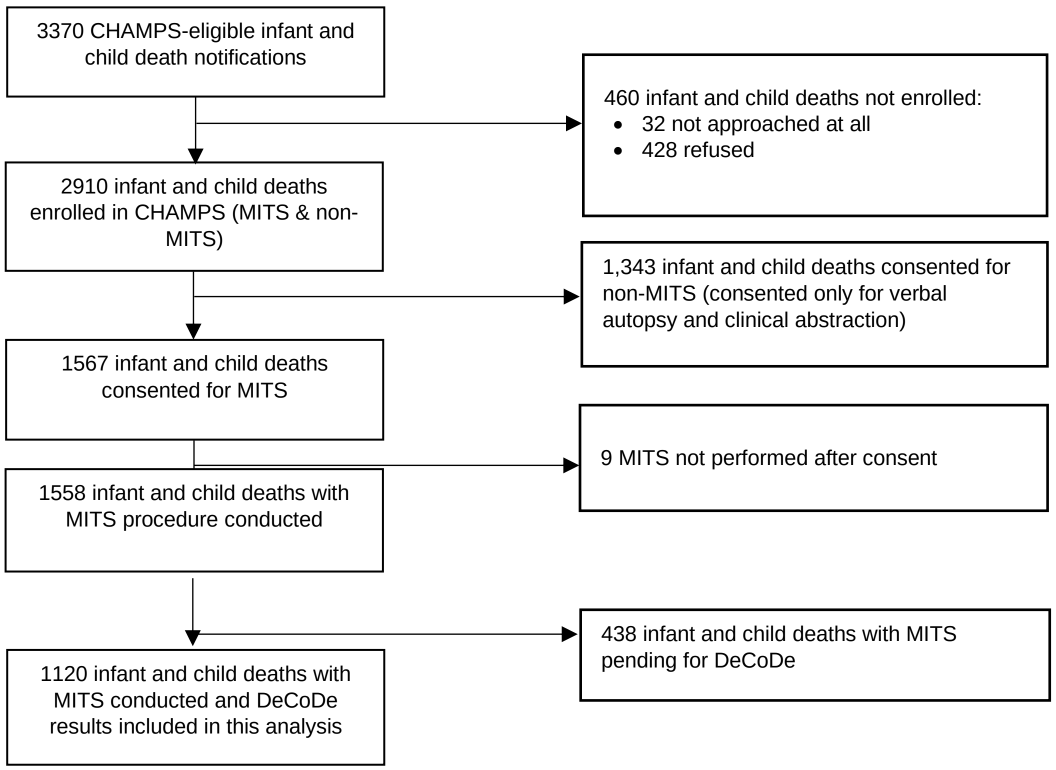

Supplemental Figure 3: Country-specific, age-group stratified childhood deaths with (Pneumonia+) or without (Pneumonia-) attributed in the causal pathway to death.

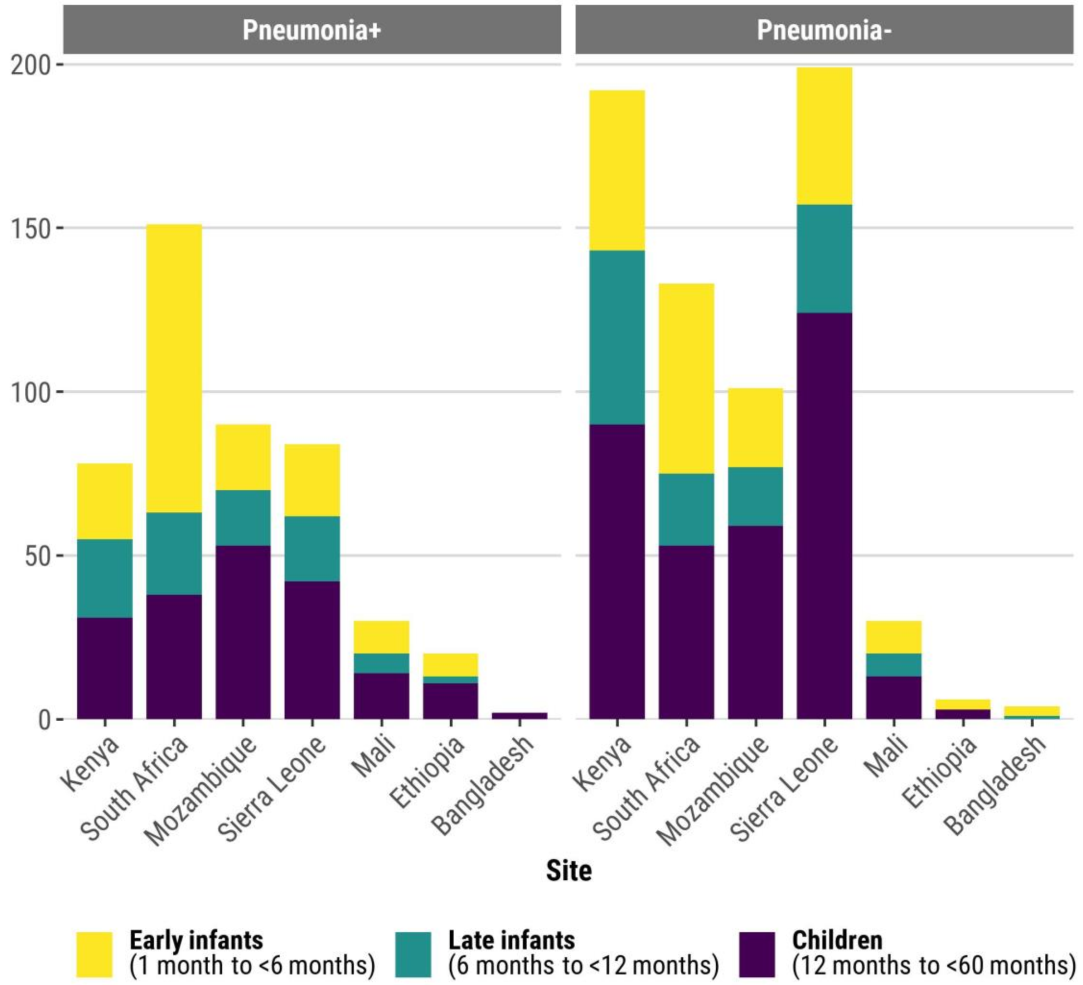

Supplemental Figure 4: Proportion of deaths determined to have died from a viral infection, bacterial infection, fungal infection, or co-infection for all pneumonia deaths and by whether the pneumonia death 1) occurred in the community or with fewer than 72 hours in the hospital or 2) or 72 or more hours after hospital admission, CHAMPS Network, December 16th 2016 to December 31st, 2022.

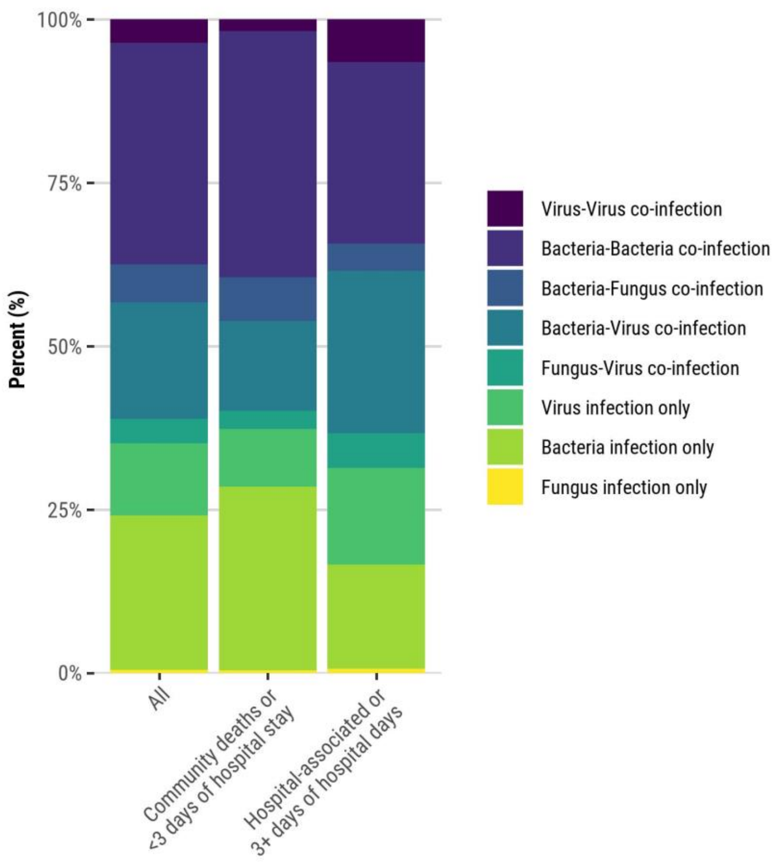

Supplemental Figure 5. Frequency of co-infections for each pathogen pair attributed to pneumonia, CHAMPS Network, December 16th 2016 to December 31st, 2022.

Panel A includes deaths that occurred in the community or with fewer than 72 hours in the hospital (N=306); Panel B includes deaths which occurred 72 or more hours after hospital admission (N=149).

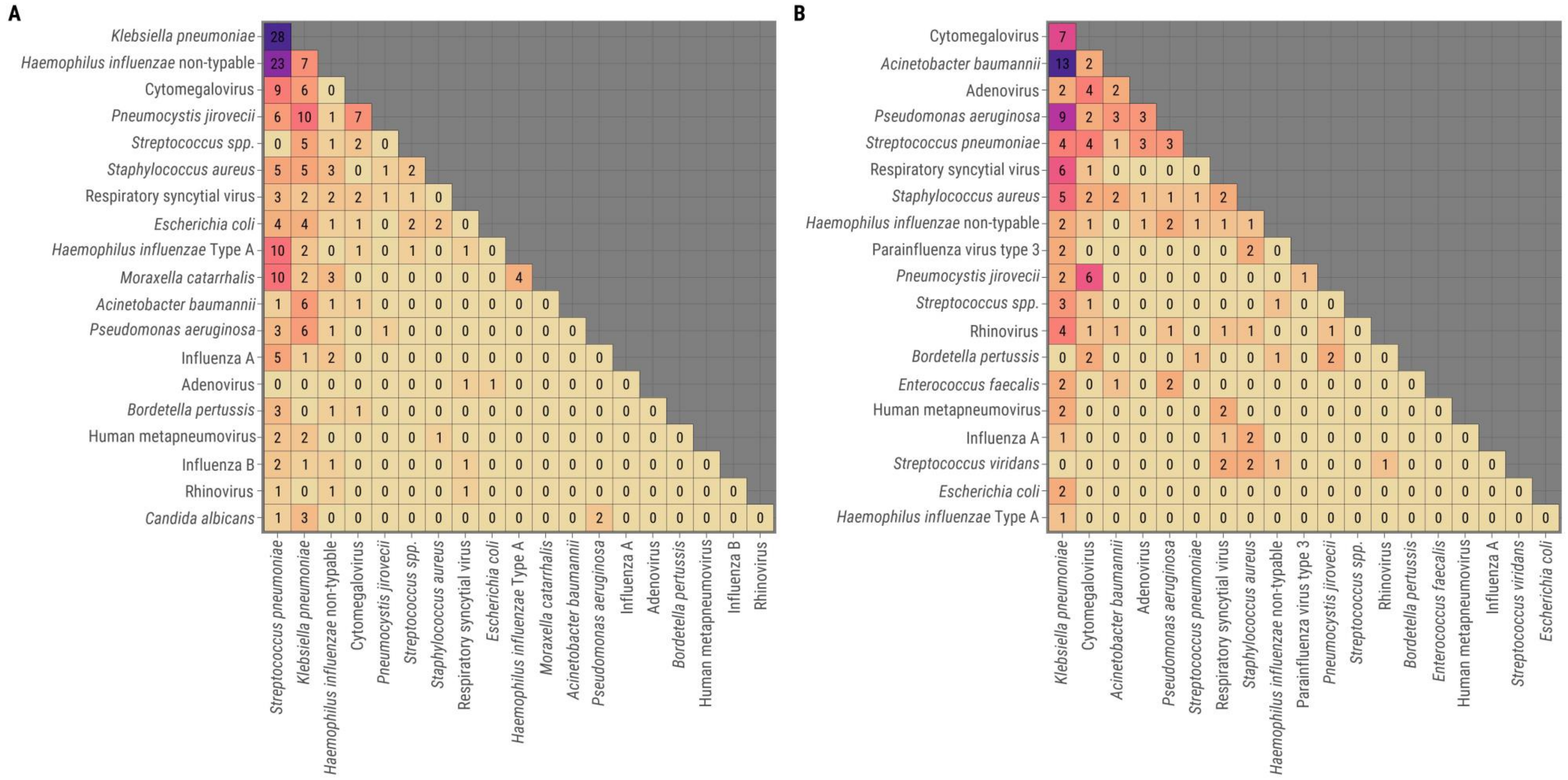

Note: Supplemental Figure 5 includes all pathogens implicated for both the underlying and antecedent pneumonias for the same death.

Supplemental Figure 6: Expert (DeCoDe) panel determination of whether pneumonia deaths were preventable (Figure 6a) and recommended improvements that could prevent such deaths (Figure 6b).

Supplemental Figure 6a

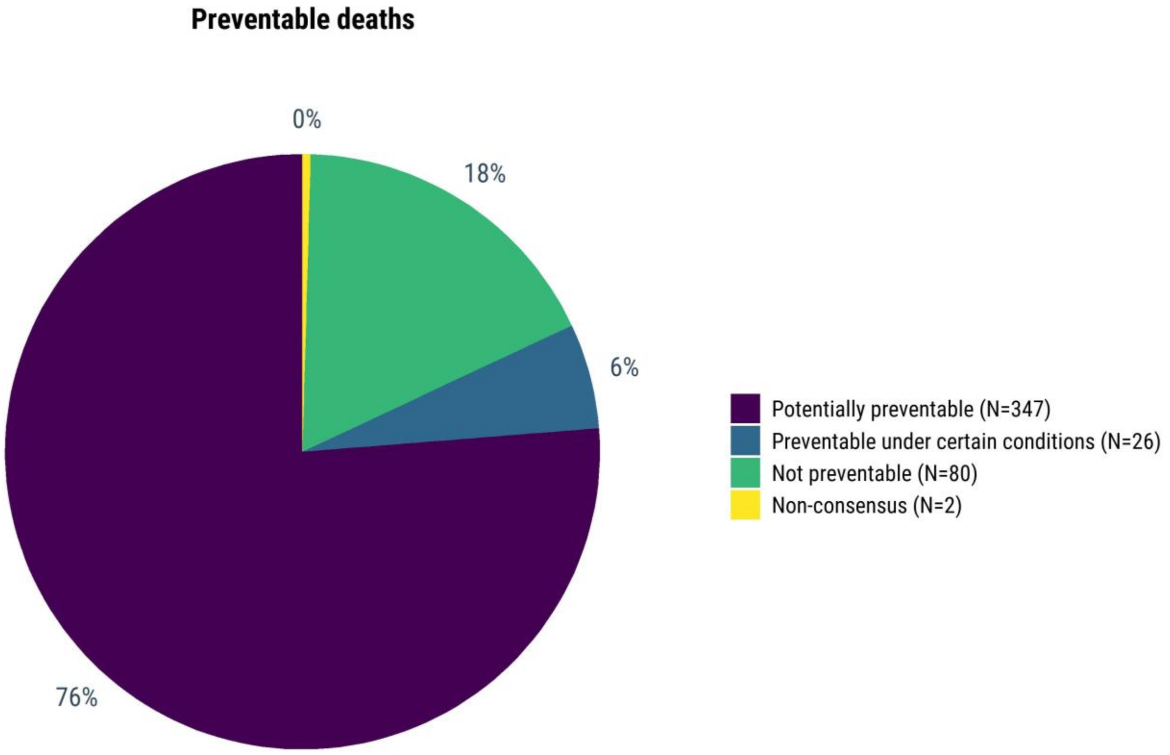

Supplemental Figure 6b

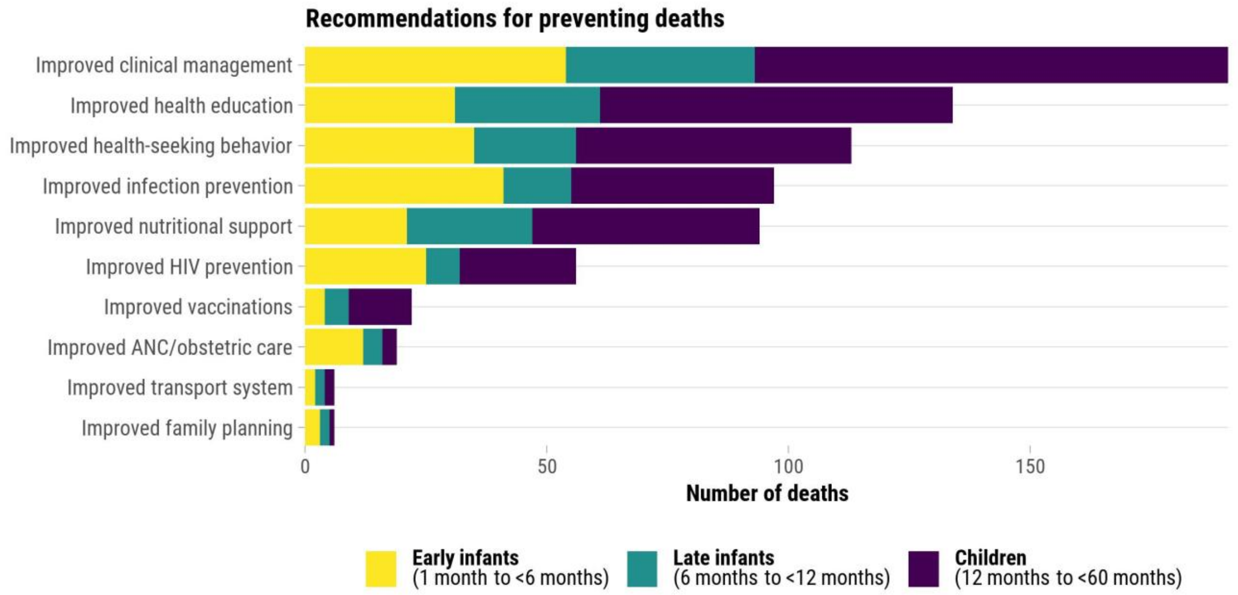

Supplemental Results.

Overall, 51·0% (n=232) of pneumonia deaths were attributed to at least one Gram-negative organism and 40·0% (n=182) to Gram-positive organisms (Table 2). There were 159 deaths with only a single pathogen attributed in the pathogenesis of pneumonia-associated death, with *K. pneumoniae* 37 (23·3%) and *S. pneumoniae* 34 (21·4%) being the most common; . Concomitant Gram-negative and Gram-positive pathogens were implicated in 21·8% (n=99/455) of pneumonia-related deaths. Furthermore, 31·9% (n=145) of deaths were attributed to at least one virus and 7·7% (n=35) to at least one fungal pathogen. The most common bacterial pathogens attributed to causing pneumonia deaths were *Streptococcus pneumoniae* (27·0%; 123/455) and *Klebsiella pneumoniae* (31·2%, 142/455). Among the 218 pneumonia deaths with more than one attributable pathogen, common co-infections included *K. pneumoniae* with *S. pneumoniae* (14·7%, 32/218), *S. pneumoniae* with non-typable *Haemophilus influenzae* (NTHi; 11·0%, 24/218), *Acinetobacter baumannii* with *K. pneumoniae* (8·7%, 19/218), and *Pseudomonas aeruginosa* with *K. pneumoniae* (6·9%, 15/218); Supplementary Figure 5. Cytomegalovirus (CMV, 11·9%; n=54), respiratory syncytial virus (RSV, 6·4%, n=29), adenovirus (5·5%; n=25) and influenza virus A/B (3·7%; n=17) were the most prevalent viruses (Figure 2). *Pneumocystis jirovecii* was implicated in the pathogenesis of 5·9% (27/455) of pneumonia deaths. Viral and fungal attributions for pneumonia deaths were more common in early infancy (52·4%; 89/170) and late infancy (39·4%; 37/94) than in childhood (27·1%; 54/199; Table 2). Among decedents with pneumonia-associated deaths with CMV as a pathogen, 48·1% (26/54) were HIV positive and 68·5% (37/54) were underweight or malnourished. In *P. jirovecii* associated pneumonia deaths, ~~and~~ 44·4% (12/27) were HIV positive and 66·7% (18/27) were underweight or malnourished.

CHAMPS Consortium

Post-mortem Investigation of Pneumonia Deaths in Children 1-59 Months in sub-Saharan Africa and South Asia countries: An observational study from 2016 to 2022,

Manuscript reference number: THELANCETCHILDADOL-D-23-00671

**First, M.I. or Middle Name**	**Surname**
Yasmin	Adam
Janet	Agaya
A.S.M. Nawshad Uddin	Ahmed
Dilruba	Ahmed
Addisu	Alemu
Solomon	Ali
Soter	Ameh
George	Aol
Solveig	Argeseanu
Farida	Ariuman
Oluseyi	Balogun
Sanwarul	Bari
Margaret	Basket
Ferdousi	Begum
Manu	Bhandari
John	Blevins
James	Bunn
Courtney	Bursuc
Carrie Jo	Cain
Richard	Chawana
Kiranpreet	Chawla
Cornell	Chukwuegbo
Kounandji	Diarra
Tiéman	Diarra
Maureen	Diaz
Babatunde	Duduyemi
Karen D.	Fairchild
Meerjady Sabrina	Flora
Ashleigh	Fritz
Mischka	Garel
Brigitte	Gaume

Mahlet Abayneh	Gizaw
Nelesh P.	Govender
Carol L.	Greene
Tadesse	Gure
Binyam	Halu
Mahbubul	Hoque
Cleopas	Hwinya
Alexander M.	Ibrahim
Kitiezo Aggrey	Igunza
Ferdousi	Islam
Okokon	Ita
Amara	Jambai
J. Kristie	Johnson
Jane	Juma
Erick	Kaluma
Mohammed	Kamal
Osman	Kaykay
Sartie	Kenneh
Sammy	Khagayi
Rima	Koka
Diakaridia	Kone
Jeffrey P.	Koplan
Nana	Kourouma
Dickens	Kowuor
Kristin	LaHatte
Sanjay G.	Lala
Kyu Han	Lee
Lucy	Liu
Hennie	Lombaard
Maria	Maixenchs
Zara	Manhique
Margaret	Mannah
Roosecelis	Martines
Ronald	Mash
Ashka	Mehta
Clara	Menéndez
Thomas	Misore
Sibone	Mocumbi
Andrew	Moseray
Francis	Moses
Christopher	Muga
Khátia	Munguambe

Nellie	Myburgh
Shailesh	Nair
Pedzisai	Ndagurwa
Ariel	Nhacolo
Tacilta	Nhampossa
Princewill	Nwajiobi
Christine	Ochola
Richard	Oliech
Bernard	Oluoch
Uma U.	Onwuchekwa
Peter Nyamthimba	Onyango
Stian MS	Orlien
Peter	Otieno
Joseph	Oundo
Harun	Owuor
Shahana	Parveen
Karen	Petersen
Samuel	Pratt
Mahbubur	Rahman
Mohammad Mosiur	Rahman
Mustafizur	Rahman
Sarah	Raymer
Jana	Ritter
Navit T.	Salzberg
Solomon	Samura
Sulaiman	Sannoh
Doh	Sanogo
Martin	Seppeh
Tom	Sesay
Joseph Kamanda	Sesay
Tahmina	Shirin
Seydou	Sissoko
Francis	Smart
Gillian	Sorour
James	Squire
Alim	Swaray-Deen
Peter J.	Swart
Fatmata Bintu	Tarawally
Saria	Tasnim
Fikremelekot	Temesgen
Sharon M.	Tennant
Cheick Bougadari	Traore

Awa	Traore
Sithembiso	Velaphi
Kurt	Vyas
Ashutosh	Wadhwa
Jeannette	Wadula
Jessica	Waller
Valentine	Wanga
Shamta	Warang
Joyce Akinyi	Were
Tais	Wilson
Jonas	Winchell
Amy	Wise
Jakob	Witherbee
Melisachew Mulatu	Yeshi
K.	Zaman
